# The evolution of the ribosome biogenesis pathway from a yeast perspective

**DOI:** 10.1093/nar/gkt1137

**Published:** 2013-11-14

**Authors:** Ingo Ebersberger, Stefan Simm, Matthias S. Leisegang, Peter Schmitzberger, Oliver Mirus, Arndt von Haeseler, Markus T. Bohnsack, Enrico Schleiff

**Affiliations:** ^1^Institute for Cell Biology and Neuroscience, Goethe University, Frankfurt 60438, Germany, ^2^Center for Integrative Bioinformatics, Max F Perutz Laboratories, University of Vienna, Medical University of Vienna, Vienna 1030, Austria, ^3^Institute for Molecular Biosciences, Goethe University, Frankfurt 60438, Germany, ^4^Faculty of Computer Science, University of Vienna, Vienna 1030, Austria, ^5^Cluster of Excellence Macromolecular Complexes, Goethe University, Frankfurt 60438, Germany, ^6^Department of Biochemistry I, Universitätsmedizin Göttingen, Göttingen 37073, Germany and ^7^Center of Membrane Proteomics, Goethe University, Frankfurt 60438, Germany

## Abstract

Ribosome biogenesis is fundamental for cellular life, but surprisingly little is known about the underlying pathway. In eukaryotes a comprehensive collection of experimentally verified ribosome biogenesis factors (RBFs) exists only for *Saccharomyces cerevisiae*. Far less is known for other fungi, animals or plants, and insights are even more limited for archaea. Starting from 255 yeast RBFs, we integrated ortholog searches, domain architecture comparisons and, in part, manual curation to investigate the inventories of RBF candidates in 261 eukaryotes, 26 archaea and 57 bacteria. The resulting phylogenetic profiles reveal the evolutionary ancestry of the yeast pathway. The oldest core comprising 20 RBF lineages dates back to the last universal common ancestor, while the youngest 20 factors are confined to the Saccharomycotina. On this basis, we outline similarities and differences of ribosome biogenesis across contemporary species. Archaea, so far a rather uncharted domain, possess 38 well-supported RBF candidates of which some are known to form functional sub-complexes in yeast. This provides initial evidence that ribosome biogenesis in eukaryotes and archaea follows similar principles. Within eukaryotes, RBF repertoires vary considerably. A comparison of yeast and human reveals that lineage-specific adaptation via RBF exclusion and addition characterizes the evolution of this ancient pathway.

## INTRODUCTION

**Figure 1.**
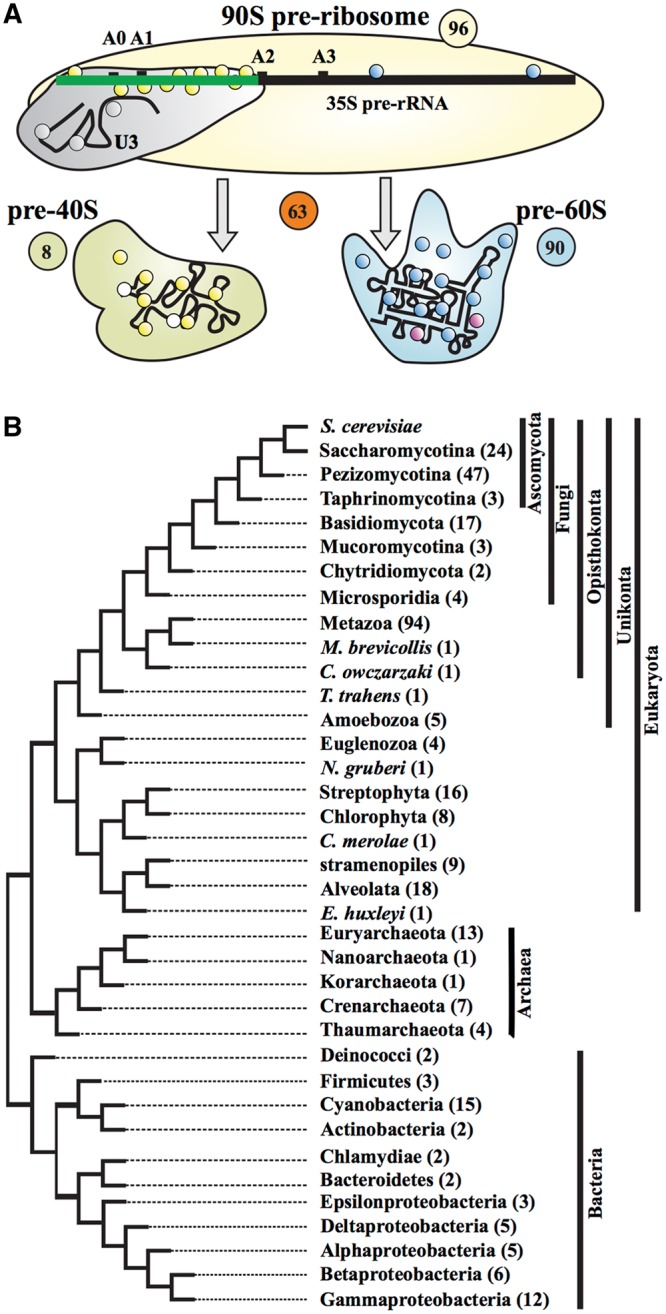
A schematic view of ribosome biogenesis in eukaryotes. (**A**) In total, 255 putative yeast RBFs were sorted according to their recruitment to the 90S pre-ribosomal complexes (96), the pre-60S (90) or the pre-40S particles (8). Two factors are represented twice as they are involved both in the formation of the 60S and 40S pre-ribosomal complexes. The 63 putative RBFs annotated as nucleases, TRAMP components or ‘not assigned’ in Supplementary Table S1 include proteins involved in RNA quality control and turnover as well as candidates that have not yet been unambiguously assigned to any complex. (**B**) The tree depicts the phylogenetic relationships of the supertaxa that we screened for the presence of yeast RBFs. The number of species subsumed in each supertaxon is given in parenthesis.

Ribosomes mediate the translation of messenger RNAs into the corresponding amino acid sequences. The biosynthesis of ribosomes is, therefore, an essential process for all living organisms. A highly complex interaction of a multiplicity of non-ribosomal proteins and small nucleolar RNAs (snoRNAs) facilitates ribosome formation (1–4). Ribosome synthesis is initiated by the transcription of a ribosomal RNA precursor (pre-rRNA; 35S pre-rRNA in yeast) in the nucleolus followed by the assembly of the 90S pre-ribosome (Figure 1A). The biogenesis pathways of the small and the large ribosomal subunits are then separated upon cleavage of the pre-rRNA transcript (at the site A2 in yeast). Most of the subsequent pre-rRNA processing events and the recruitment of the independently transcribed 5S rRNA occur in the nucleus. The resulting pre-40S and pre-60S ribosome subunits are then exported into the cytoplasm where the final rRNA processing takes place [for yeast, see (5,6)]. Eventually, both ribosomal subunits undergo final maturation to become functional for translation (1,6).

Among eukaryotes, the pathway is understood best in the yeast *Saccharomyces cerevisiae* (1,3,4). Sets of pre-ribosomal intermediates have been isolated via tandem affinity purification (7–10). Their analysis revealed more than 200 non-ribosomal proteins with diverse biochemical activities. This includes putative RNA helicases, multiple GTPases, ATPases and RNA-binding proteins, as well as endo- and exonucleases (1,11–13). A number of these proteins apparently act as stand-alone factors in ribosome biosynthesis. For example, individual proteins presumably mediate most of the endonucleolytic cleavages. Other proteins are already known to interact in functional complexes. For example, one of the early nucleolar pre-rRNA processing steps and the 5.8S rRNA biogenesis require the yeast RNase MRP, a complex comprising a RNA component and at least 10 different proteins (14,15). Similarly, the exosome complex mediates 3′–5′ exonucleolytic trimming in various processing steps, partially in conjunction with the TRAMP complex that harbors the poly(A) polymerase Trf4 (16). The modifications of the rRNA required for translation accuracy and fidelity are guided by snoRNAs that form base pairs with the corresponding regions in the pre-rRNAs. Seventy-five snoRNAs in yeast act as part of box C/D or box H/ACA snoRNPs that perform methylation and pseudouridylation reactions, respectively (17–19). The binding sites of most snoRNPs on pre-rRNA have been verified in yeast. In contrast, the sites of action and the molecular functions of only a small subset of the proteinaceous ribosome biogenesis factors (RBFs) have been analyzed. This is mainly due to the high complexity of the ribosome biogenesis pathway and the large number of components involved.

Compared to *S. cerevisiae*, much less is known about the ribosome biogenesis pathway in animals or plants. *In silico* tracing of yeast RBFs in other species concentrated on individual sub-complexes such as the RNase MRP (20,21) or few ribosome export factors (22). It was only recently that an initial inventory of factors involved in ribosome biogenesis in human cells was proposed (23). And besides studies of plant snoRNAs and their genes (24,25), only few components of the plant ribosome biogenesis pathway have been identified and investigated (26–32). Thus, an exhaustive analysis of eukaryotic RBF repertoires is still missing. Insights into ribosome biogenesis of the archaea are even more limited. Research in the sister domain of the eukaryotes (33,34) has mainly focused on snoRNP complexes [reviewed in (2)] and on individual RBFs (35,36). Although the few identified factors do have eukaryotic counterparts, it remains unclear if and to what extent the general concept of ribosome biogenesis in archaea resembles that of the eukaryotes.

The apparent gap in knowledge concerning differences and similarities in ribosome biogenesis across species, and concerning the evolution of this pathway is contrasted by the wealth of data available. Whole genome sequencing projects in all three domains of life have determined the sets of protein-coding genes for a large number of species (see http://www.diark.org). Tools abound to search for orthologs in this data (37–39). From the resulting presence–absence patterns for a particular protein across the analyzed species, i.e. its phylogenetic profile, it is possible to approximate its evolutionary age. Genes of the same evolutionary age can then be summarized in so-called phylostrata (40,41), and it was eventually shown that functionally interacting genes within one phylostratum provide information about when in evolutionary history the corresponding pathway emerged (40). However, there is a conceptual problem in this approach, namely the considerably weak link between orthology of two proteins and their functional equivalence (42). More precisely, the sheer presence of an ortholog to a protein of interest does not necessarily indicate that the proteins function is also conserved. Moreover, the severity of the problem increases the farther the phylogenetic distance between the analyzed species, as the corresponding proteins had more time to evolve to different functionalities. Thus, relying on orthology prediction alone poses the risk of substantially overestimating the phylogenetic distributions and evolutionary ages of functional pathways. This calls for the consideration of further evidences, even in large scale phylogenetic profiling studies, as they result, e.g. from the analysis of functional protein domains (43) or even of entire feature architectures (44) to strengthen the assumption that two orthologs share the same function.

Here, we have set out to systematically investigate the evolutionary history of ribosome synthesis from a yeast perspective. We have integrated ortholog searches for 255 non-ribosomal yeast RBFs in 261 eukaryotes, 26 archaea and 57 bacteria with Pfam domain architecture analyses to generate a comprehensive phylogenetic profile of the corresponding pathway. RBF candidates identified outside the eukaryotes were subjected to manual curation using FACT (44) to judge whether or not they are likely to convey the same activity as their yeast orthologs. The resulting inventories of RBF candidates for 344 species form now a solid basis for investigating ribosome biogenesis across the tree of life.

## MATERIALS AND METHODS

### RBF factor selection

We selected proteins supposedly involved in yeast ribosome biogenesis by extensive literature search [cited in the introduction and (45)]. In the first round, all factors associated with any stage in the biogenesis process were collected. The assignment to a particular stage was subsequently specified by additional literature search such that each factor was only assigned to the earliest biogenesis stage it was found to be involved in. Factors involved in RNA quality control or turnover, as well as proteins with only hypothetical involvement in ribosome biogenesis were grouped separately. The final set comprised 255 yeast proteins and an overview is given in Supplementary Table S1.

### Sequence data

We analyzed protein-coding sequence data inferred from the completely sequenced genomes of 261 eukaryotes, 26 archaea and 57 bacteria. The species names and data sources are provided in Supplementary Table S2.

### Identification of orthologs

We identified orthologs for the 255 yeast RBFs using HaMStR-OneSeq, an extended version of the HaMStR approach (39). For each of the 255 query proteins, HaMStR-OneSeq first compiled in an iterative procedure automatically a core-set of orthologous groups together with the corresponding reference taxon sets. Both sets in conjunction were then used for the final ortholog search.

*Iterative compilation of the core ortholog groups.* In iteration 1, each core ortholog group was initialized with the query protein from yeast and subsequently transformed into a profile hidden Markov model (pHMM) with hmmbuild version 3 (http://hmmer.janelia.org). The set of reference taxa was initialized with yeast. A HaMStR search with the initial pHMM was then started in the protein sets of 344 species with completely sequenced genomes. The resulting candidate orthologs were locally aligned to the query protein, and the highest scoring protein was added to the core ortholog group. The corresponding taxon was added to the reference taxon set. The iterations 2 to 5 consisted of the following steps: the proteins in the core ortholog group were aligned with mafft v. 6.0 using the most sensitive option L-INS-i (46). The updated alignment was used to re-train the pHMM, and a new HaMStR search was performed in all taxa that were not already in the reference taxon set. The HaMStR output was then filtered by removing sequences from all species whose genus is already represented in the reference taxon set. This step increased the phylogenetic diversity within the core ortholog set. The remaining candidates were then pair-wise aligned against all sequences in the core ortholog group, and the one with the highest average alignment score across all pair-wise alignments was added. This concluded the iteration. After five iterations, the sequences in the core ortholog group were aligned with mafft (46), and a final pHMM was trained. Note, that the benchmarking of HaMStR revealed that core ortholog groups consisting of five sequences are sufficient for an accurate ortholog prediction (39).

*Ortholog search.* For each of the 255 yeast RBFs, we used the automatically generated core ortholog group together with the corresponding pHMM and performed a HaMStR search in 344 taxa. Yeast was used as the reference taxon. In the case that HaMStR predicted two or more candidates in a given species, we aligned each candidate against the corresponding yeast RBF and identified the highest scoring protein as representative. Lower ranking candidates were accepted as co-orthologs when their pair-wise distance to the representative ortholog was smaller than the distance between the representative ortholog and the yeast RBF (37). The procedure generated for each RBF a collection of putative orthologs and the full set of orthologs can be downloaded from: http://www.deep-phylogeny.org/rbg/rbf-orthologs-full.fa.gz.

### Analysis of protein domains and feature architectures

We annotated Pfam (43) domains in the yeast proteins and their predicted orthologs with hmmscan from the HMMER3 package (http://hmmer.janelia.org). Additional sequence features were annotated using the feature architecture comparison tool FACT (44). Comparisons of the feature architectures between pairs of proteins, feature architecture-based similarity searches and manual curation of the ortholog candidates were done via the FACT web pages (http://fact.cibiv.univie.ac.at).

### Manual curation of candidates

The manual curation of RBF orthologs served two purposes. First, we validated the ortholog assignments in questionable cases, i.e. when the phylogenetic profile indicated multiple and independent losses of a gene. Second, we assessed whether or not the yeast protein and its ortholog are likely to share the same biochemical activity. We applied the following bipartite procedure. In step 1, we used the candidate ortholog as query for a BLASTP search in the yeast protein set. We then determined the extent of sequence similarity (BLAST bit score) between the query and the best hit—the yeast RBF—and between the query and the BLAST hits on ranks 2 to maximally 5. The confidence in the ortholog assignment increased with increasing bit score of the best hit and with an increasing score difference between the best hit and the lower ranking hits. Note, we did not use any hard thresholds and rather evaluated each candidate case-by-case. In step 2, we determined the feature architecture of the candidate ortholog, i.e. its linear arrangement of, e.g. functional domains (Pfam and Smart), transmembrane domains, secondary structure elements and low complexity regions. We then used FACT (44) to score the feature architecture similarities between the candidate and all 6697 yeast proteins. The yeast proteins were then ranked according to the FACT score, and the support for a candidate increased the higher the rank of its orthologous yeast RBF was, and the fewer proteins achieved a comparable score. Eventually, we inspected the feature dotplots by eye to judge the overall similarity of two proteins apart from any score. Criteria were here the extent to which two proteins agree in overall length, but also length and order and type of shared features.

Based on the outcome of the previous steps, we assigned four levels of confidence. A ‘level-1’ (trust) candidate identifies the yeast RBF as a unique best BLAST hit and also as the protein with a top or at least high-ranking feature architecture similarity. A ‘level-2’ (possible) candidate can have either one to few lower ranking BLAST hits with scores comparable to that of the best BLAST hit, or its feature architecture matches that of other yeast proteins slightly better. A ‘level-3’ (questionable) candidate has typically multiple BLAST hits with scores comparable to that of the best BLAST hit and the feature architecture similarity does not clearly vote for the yeast RBF as the most similar protein. All other candidates were assigned as ‘level-4’ (not trust). Note, that the assignment of the confidence levels includes also the visual inspections of the feature dotplots. Thus, in individual cases we may have up- or downgraded a candidate depending on personal judgment. To make the decisions reproducible, we provide for all curations links to the FACT/BLAST results.

### Identification of paralogs within yeast RBFs

Individual yeast proteins that originated by a duplication of a single ancestral gene share part of their evolutionary history. To identify such within-species paralogs among our yeast RBFs we followed a simple rationale. If two yeast RBFs are evolutionarily related we should detect them as co-orthologs to a protein of a second species that split from the yeast lineage prior to the gene duplication event. Choosing the second species in order of increasing evolutionary distances to yeast will then indicate if any two yeast RBFs are related and when the corresponding gene duplication at latest must have taken place. Note that this procedure is both highly sensitive and has a very low false positive rate (47). More precisely, we proceeded as following. We screened InParanoid orthologous groups between yeast and species with increasing evolutionary distance as deposited in the InParanoid database (48). In instances where two yeast RBFs occurred in the same orthologous group we flagged them as paralogous and dated the corresponding gene duplication event after the split of the respective species and yeast. As no archaea are represented in the InParanoid database we carried out InParanoid orthology prediction between yeast and all 26 archaean species (Supplementary Table S2) locally. In a complementary approach, we searched the RBFs for proteins with the same Pfam domain composition. The only resulting candidates that were not already flagged as paralogs in the first step (RIO1 and RIO2) were then subjected to maximum likelihood tree reconstruction to confirm their evolutionary relationships. We aligned the corresponding sequences with mafft (46). Alignment columns with more than 50% gaps were removed with an in-house Perl script, and a maximum likelihood tree was computed with RAxML (49) using PROTGAMMAILGF.

## RESULTS AND DISCUSSION

### The phylogenetic profile of ribosome biogenesis in yeast

We set off by creating an overview of the ribosome biogenesis machinery in *S. cerevisiae*. A literature screen identified 255 accessory proteins confirmed or proposed to play a role in this pathway (Figure 1A; Supplementary Table S1). Starting from this set we investigated the evolutionary ancestry of ribosome biogenesis in yeast. We determined the initial phylogenetic profile of the 255 yeast RBFs by searching for their orthologs in 261 completely sequenced eukaryotes, 26 archaea and 57 bacteria (Supplementary Table S2). The pair-wise orthology predictions between yeast and each of the 344 species were performed with HaMStR-OneSeq, an extended version of the HaMStR algorithm (39) and identified a total of 64 528 orthologs in 51 537 distinct orthologous groups (available at http://www.deep-phylogeny.org/rbg). On average, each eukaryotic species harbors orthologs to 190 yeast RBFs, yet the range within eukaryotes is substantial. For example, *Saccharomyces paradoxus* exhibits almost the full inventory of 254 RBFs, in the microsporidium *Antonospora locustae*, which is still considerably closely related to the fungi we detected orthologs to only 65 RBFs, and in humans we found again counterparts to 200 yeast RBFs. For the archaea and bacteria, the values are substantially smaller, with a mean of 31 and 18 RBFs, respectively. If we focus on individual RBFs we see a similar variation of which MMP6 and DIM1 are the most extreme examples. They are represented by orthologs in 6 (∼2%; MPP6) and 341 (∼99%; DIM1) species, respectively.

**Figure 2.**
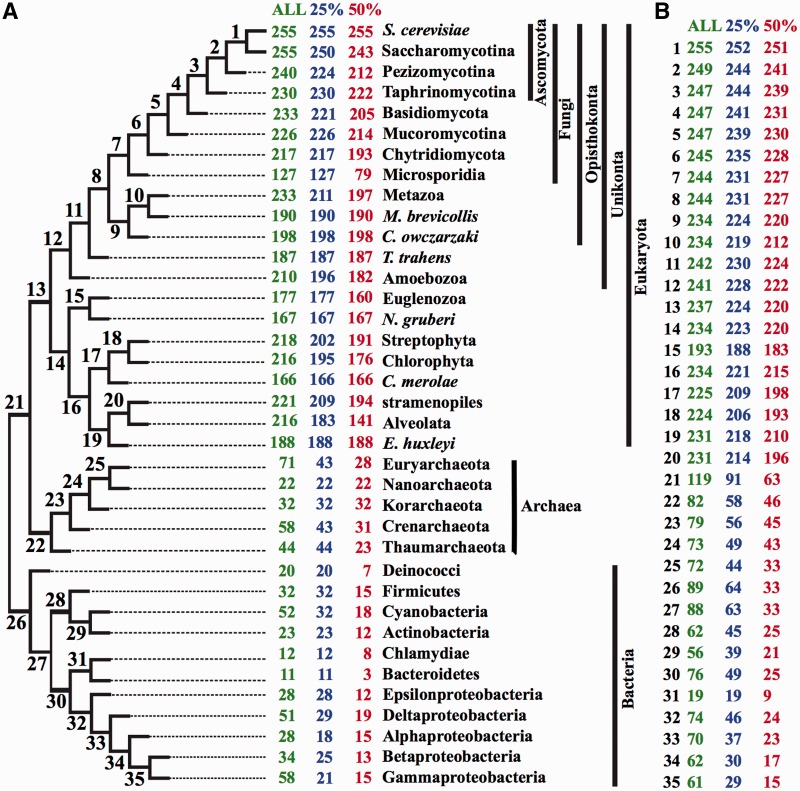
RBF ortholog sets in the three domains of life. (**A**) We have searched for orthologs to the 255 putative yeast RBFs in 36 supertaxa comprising 344 species. The number of RBFs for which an ortholog was identified in at least 1 (ALL), 25% and 50% of the subsumed species are given next to the supertaxon name. (**B**) Ancestral numbers of RBFs for the internal nodes of the tree. The numbers in black correspond to the node labels of the tree depicted in (A).

To obtain a more comprehensive view on the evolutionary history of ribosome biogenesis the data were put into a phylogenetic context. We reduced the complexity of this analysis by summarizing the 344 species into 36 monophyletic groups (supertaxa) representing major clades in the tree of life (Supplementary Table S2). We arranged these supertaxa together with yeast in a three domains tree of life (33,34) grouping the eukaryotes according to the results of Derelle and Lang (50), the archaea according to Spang *et al.* (51) and the bacteria according to Toft and Andersson (52) (Figure 1B). We then determined for each of the 255 yeast RBFs whether or not it is represented by an ortholog in the individual supertaxa (Figure 2A). In the most permissive setting, we considered a yeast factor as represented in a supertaxon when an ortholog was detected in at least one of the subsumed species. In the more stringent settings an ortholog must be detected in more than 25% and 50% of the subsumed species, respectively. The analysis at various stringencies gives an impression of how the orthology predictions in individual or few species drive the outcome for the whole supertaxon. This is particularly relevant for the supertaxa comprising many species, as the risk for accepting a spurious ortholog increases with the number of proteins compared. Our results reveal only a weak correlation between phylogenetic distance of a supertaxon to yeast and the number of orthologs detected (Figure 2A). As an example, we observe at the lowest stringency level orthologs to 240 RBFs in the Pezizomycotina, the closest relatives of the Saccharomycotina. In animals (Metazoa), the sister group to the fungi, we still find orthologs to 233 RBFs, and in land plants (Streptophyta) orthologs to 218 RBFs. Only when we move to the archaea and bacteria we observe a marked decrease. In archaean clades only up to 71 yeast RBFs are represented by an ortholog (Euryarchaeota) and in bacterial clades a maximum of 58 can be found (γ-proteobacteria). At the two levels of higher stringency the absolute numbers of yeast RBF represented in the individual supertaxa are reduced but otherwise the trend remains unchanged.

### General comparison of the inventory of ribosome biogenesis factors

To assess the plasticity of the RBF set through time we inferred the size and composition of the ancestral sets at the internal nodes of our tree using Dollo parsimony (53). In other words, we assumed that the detection of an ortholog to a yeast RBF in a non-yeast species implies that this RBF was present in the common ancestor of the two species (Figure 2B). Notably, the set of RBFs are strikingly stable when following the tree from yeast backwards in time. Of the 255 RBFs we can trace back 237 to the last common ancestor of all eukaryotes (node 13 in Figure 2B). This number only slightly reduces to 220 when using the most stringent 50% option. Note, that we find almost the same numbers at various other nodes in the eukaryotic subtree, e.g. for the common ancestors of animals and their closest relatives (node 9: 234), of plants and green and red algae (node 17: 225) and of alveolates and stramenopiles (node 20: 231). This indicates that our findings are by and large robust to changes of the taxon sampling and of the tree topology. In summary, our results so far suggest that ribosome biogenesis, as it exists nowadays in yeast, is an evolutionary ancient process, where the basic principle was laid out already in the last common ancestor of all eukaryotes. However, our findings also point to the existence of an evolutionary even older set consisting of genes, which predate the prokaryote–eukaryote split.

*Lineage-specific loss of RBF genes. *Based on the reconstruction of the ancestral RBF inventories (Figure 2B), we can now determine the extent of apparent gene loss along the tree. However, six of our eukaryotic supertaxa are represented only by a single species (*Cyanidioschyzon merolae*, *Monosiga brevicollis*, *Capsaspora owczarzaki*, *Thecamonas trahens*, *Emiliania huxleyi* and *Naegleria gruberi*) and only draft genome assemblies are available. Therefore, it is not clear whether in these cases the missing genes are truly absent or whether the corresponding gene loci have not yet been annotated or sequenced. Among the remaining eukaryotic supertaxa the most remarkable amount of gene loss is seen in the Microsporidia. Of the 244 RBFs present in the common ancestor shared with the fungi [Figure 2B; node 7, (54,55)] only about half are retained in contemporary Microsporidia. A similar albeit not quite as extensive loss is seen on the branch leading to the contemporary Euglenozoa/Heterolobosea. Here, we find a maximum of 177 and 167 factors, respectively, suggesting a loss of more than 60 genes. Yet, both Microsporidia and Euglenozoa are renown for their accelerated rate of protein sequence evolution (54,56). Thus, we possibly miss a substantial number of orthologs as sequence similarity may not suffice for their detection at our level of stringency. To address this issue, we performed a second, manual search exemplarily in the microsporidium *Encephalitozoon cuniculi*. Using both BLAST (57) and a complementary feature architecture based search (44), we screened the *E. cuniculi* protein set for traces of the 128 yeast RBFs so far missing in Microsporidia. As a result we found only six additional candidates (Table 1; Supplementary Text 1; Supplementary Table S3). Four of these lack a significant sequence similarity with their alleged counterpart in yeast. They could only be identified via their feature architecture similarity to the yeast RBF. The impact of our refined and comprehensive search for RBF candidates in *E. cuniculi* is, in terms of numbers, negligible. This suggests that the reduced RBF repertoires for Microsporidia in general are not a methodological artifact, and the same applies presumably to other fast evolving species. Instead, it demonstrates that this ancient and functionally highly relevant pathway displays some evolutionary plasticity. Still, the situation in Microsporidia is among all analyzed taxa the most extreme. This is most likely related to their adaptation to obligate intracellular parasitic lifestyle causing extensive genome compaction and accompanied gene losses (54). It is an open question if Microsporidia nonetheless build their ribosome autonomously or whether they recruit factors from their host. If the former is true then they must have evolved a substantially simplified way to produce functional ribosomes.

**Table 1.** RBF candidates in *E. cuniculi* resulting from a joined FACT and BLAST search

Yeast RBF	*E. cuniculi* candidate	Evidence^a^	BLAST bit score (e-value)
AIR1	Q8SU59	F/B	59 (4E-10)
CSL4	Q8SVT7	F	–
ECM16	Q8SR50	F/B	164 (1E-41)
ENP1	Q8STP8	F	–
POP4	Q8SUV2	F	–
RRP1	Q8SV05	F	–

^a^FACT and BLAST.

*Lineage-specific duplications of RBF genes. *Complementary to analyzing the lineage-specific loss of yeast RBFs we next concentrated on lineage-specific duplications of RBF orthologs in the individual clades. Already an initial survey of our data indicates that the impact of gene duplications is only moderate. Among the 51 537 pair-wise ortholog groups between yeast and each of the 344 species we find only 6343 instances with two or more co-orthologs in a non-yeast species. The median number of co-orthologs for the individual supertaxa and yeast RBFs are given in Supplementary Table S4. Only the Haptophycea, represented by *E. huxleyi*, possess two or more co-orthologs for more than 50% of the yeast RBFs. Even the green plants having undergone several rounds of whole genome duplications (58) maintained for 152 of the 210 RBFs a one-to-one orthology in at least half of the analyzed species. The latter observation is in line with a recent report that convergent gene loss subsequent to whole genome duplications in flowering plants has led to the (re-)formation of single copy genes (59). Looking at each of the 255 RBF individually reveals that also most RBFs occur with a median copy number of one in most supertaxa. There are few notable exceptions of which SRM1, a protein involved in nucleocytoplasmic trafficking, is the most prominent one. SRM1 occurs with a median above 2 in 11 of the 36 supertaxa, and we found up to 17 and 49 co-orthologs for the Streptophyta and *E. huxleyi*, respectively. Overall, however, a picture emerges where gene duplications have only a limited impact on the long-term evolution of the ribosome biogenesis pathway.

### Domain architectures and biochemical properties of RBF orthologs

**Figure 3.**
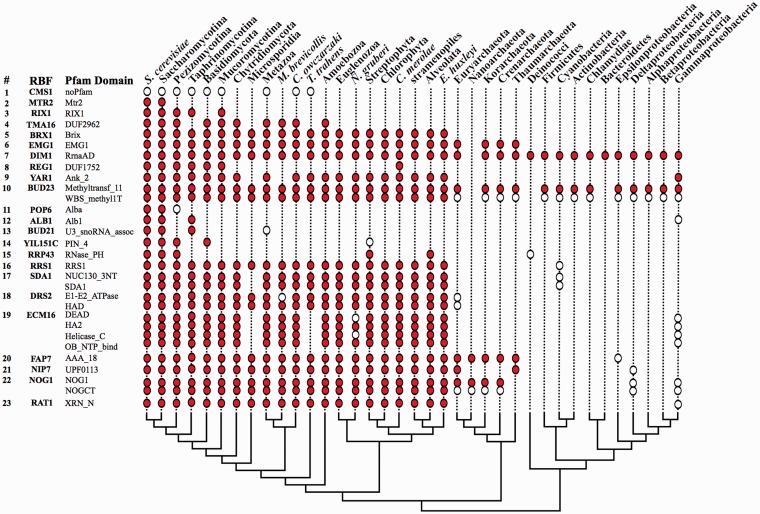
The phylogenetic profiles of yeast RBFs and their Pfam domains. The matrix summarizes a selection of representative examples, and the full data are given in Supplementary Table S5. We considered a RBF as present in a systematic group when an ortholog was identified in at least one of the subsumed species (circles). If, in addition, at least one ortholog within a supertaxon shares a given Pfam domain with the yeast RBF, the corresponding circle is filled. If the Pfam domain of the yeast RBF is absent the circles remain unfilled. The tree represents the phylogenetic relationships of the 24 taxa and yeast. Factor 1 (CMS1) is one of the 14 RBFs without a significant hit against any Pfam domain. Factors 2–7 represent examples where we identified orthologs in taxa with increasing phylogenetic distance to yeast. Factors 8 (REG1) and 9 (YAR1) have conspicuous phylogenetic profiles implying multiple independent losses. For factor 10 (BUD23) we identified orthologs in all analyzed taxa including the bacteria. However, the archaean and bacterial orthologs miss one of the two Pfam domains present in the yeast factor. The remaining examples represent all factors for which the estimate of their evolutionary age is influenced by orthologs that do not share any Pfam domain with the yeast protein.

The phylogenetic profiling of yeast RBFs via orthology prediction (Figure 2) is only the first step in tracing the evolutionary history of the corresponding pathway. Orthology is, per definition, a statement concerning the evolutionary relationships of two genes (60), and orthology assignments are made irrespective of the contemporary genes’ functions. While there is a good chance that functionally equivalent proteins are orthologous, the reverse conclusion requires careful attention (61,62). An experimental functional characterization for even a fraction of our candidates and the corresponding yeast RBFs is beyond the scope of this project. Thus, we gathered further bioinformatics evidence to support the assumption that the identified orthologs have at least the same biochemical activity as their counterparts in yeast. Note that we now differentiate between biochemical activity and function of a protein. The biochemical activity represents the reaction it catalyzes and can, to a certain extent, be predicted from the protein sequence. In contrast, protein function combines the biochemical activity and the cellular process or pathway in which this activity is embedded, which cannot be inferred from sequence analysis alone. In our analysis, we proceeded with comparing the domain architectures between the yeast RBFs and their orthologs. We annotated all Pfam (43) domains in the 255 yeast proteins. Thirteen yeast proteins did not contain any Pfam domain and further four obtained only an insignificant hit in the domain search. Subsequently, we compared the Pfam domain content between the yeast RBFs and the corresponding orthologs (Figure 3; Supplementary Table S5). We then excluded all proteins from further analysis that do not share at least one domain with their yeast ortholog. This reduced the set of RBF candidates by 1788 proteins, a number that appears on the first sight moderate. However for 10 RBFs we excluded at this step all archaean or bacterial orthologs (Figure 3), by that substantially altering their phylogenetic profile together with the resulting evolutionary age estimates.

**Figure 4.**
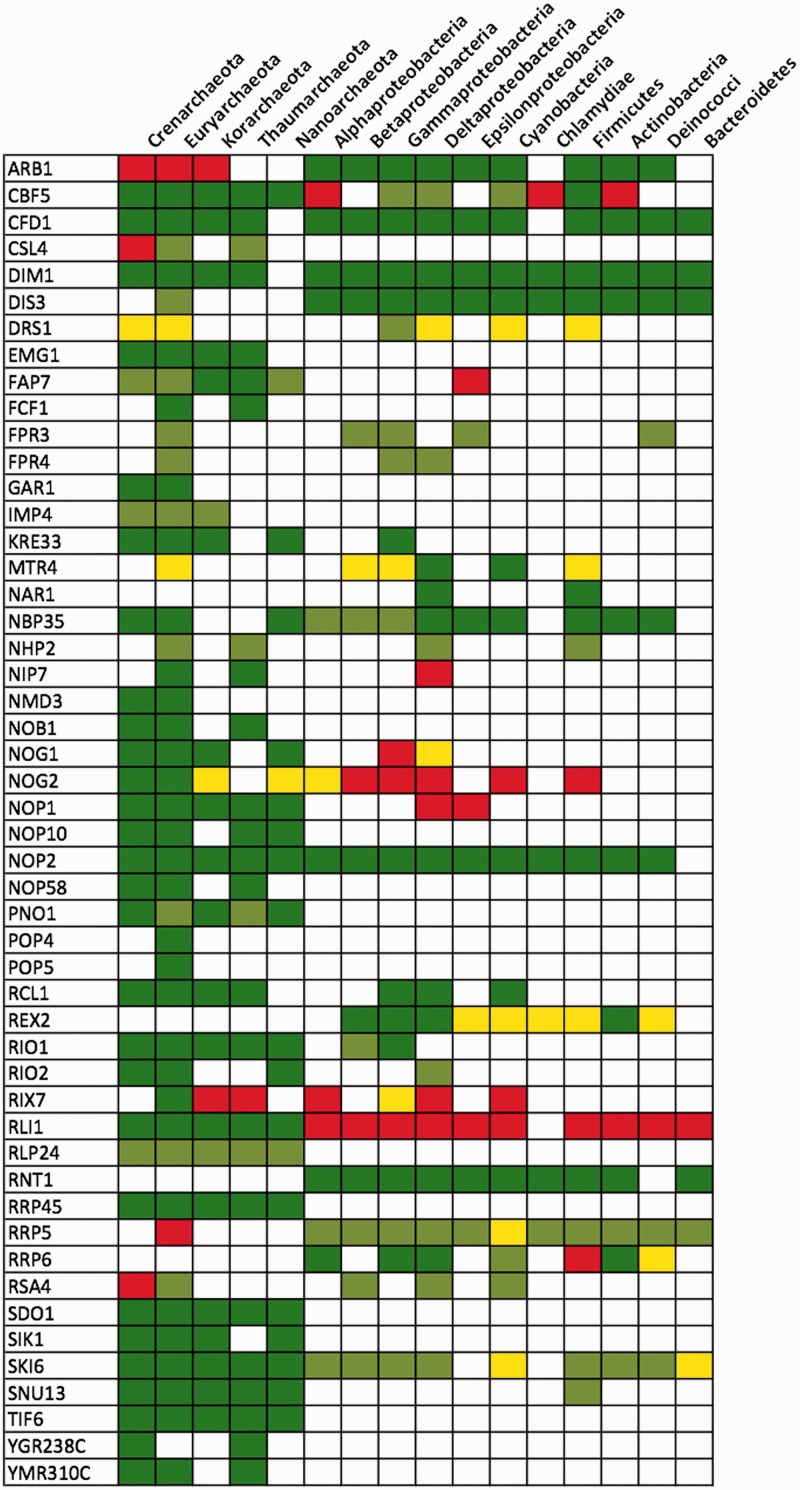
Yeast RBFs with ‘level-1/2’ candidates in archaea and bacteria. We have detected and curated orthologs to 82 RBFs in the archaea and/or the bacteria sharing at least one Pfam domain with the yeast protein. The figure represents the RBFs with at least one ‘level-1’ or ‘level-2’ candidate outside eukaryotes. The full table is given in Supplementary Table S6. Colors denote different confidence levels: dark green, ‘level-1’; light green, ‘level-2’; yellow, ‘level-3’; red, ‘level-4’.

*Manual curation of RBF candidates. *Subsequently, we extended the analysis of shared domains to a full comparison of feature architectures (44). As this procedure requires the visual inspection of every individual protein we restricted it to two critical subsets: We curated all RBF candidates identified in the archaea and the bacteria as their acceptance has a strong impact on the age estimate for the corresponding yeast RBF (Figure 4; Supplementary Table S6). In addition, we investigated all eukaryotic candidates where the phylogenetic profile indicates an excess of independent gene losses across supertaxa as this may point out false positives in the ortholog search. REG1 in the red algae *C. merolae* is an illustrative example for the latter case (Figure 3). For the curation we used the RBF candidate as query for a reverse FACT and BLAST search in the yeast proteome, respectively. We then assessed the extent of feature architecture similarity between the query and its assigned ortholog relative to the feature architecture similarity of the best FACT hit. Likewise we compared the extent of sequence similarity (reflected in the BLAST score) of the best BLAST hit (the ortholog) to that of the lower ranking hits. A candidate achieved maximal support when the best FACT hit was its ortholog in yeast, and when no other protein in the yeast proteome has a comparable sequence similarity. A recent benchmarking revealed that in such instances two proteins have the same activity in 99% of the cases (44). In total we assigned four confidence levels: level-1 candidates are judged to very likely have the same biochemical activity (trust), level-2 candidates are considered ‘possible’, level-3 candidates ‘questionable’ and level-4 candidates ‘not trustworthy’ (see ‘Materials and Methods’ section for details).

To gauge the quality of our curation we used the functional annotations of both yeast and *Escherichia coli* proteins in literature and databases (e.g. SGD at http://www.yeastgenome.org). Of the 24 proteins that we identified as RBF candidates in *E. coli* 13 were ranked as ‘level-1’, one each as ‘level-2’ and ‘level-3’ and the remainder as ‘level-4’ candidates. We extracted for these proteins and for their yeast orthologs the corresponding annotations and used them to evaluate our curation (Supplementary Text 2; Supplementary Table S7). Notably, the biochemical activities described for 11 of the 13 ‘level-1’ RBFs and of the single ‘level-2’ candidate agree with those of the yeast orthologs. For all 10 candidates of ‘level-3’ (questionable) and ‘level-4’ (not trust) the annotations differ, indicating that candidates of these levels are indeed not trustworthy.

We now briefly describe the two exceptions among the ‘level-1’ candidates. AAC75527 is the *E. coli* ortholog to KRE33 in yeast. The protein acts as an acetyltransferase modifying the anticodon of the elongator methionine tRNA [ac4C34; (63)], yet there is no indication for a participation in bacterial ribosome assembly. KRE33 in yeast is essential for biogenesis of the small ribosomal subunit, but its exact activity is still not known (Supplementary Text 2). Thus, although the two proteins clearly differ in their functionality, i.e. in the pathway they are integrated in, there is no contradiction in their annotated activity. Our analysis has revealed that the two proteins are reciprocal best hits both in the BLAST and in the FACT search with no other protein achieving comparable scores. Moreover, both proteins have almost identical feature architectures (Supplementary Figure S1A). As a consequence, there is no reason to doubt the common ancestry of the two proteins, and because they display identical functional domains it is conceivable that they also have the same biochemical activity. We therefore propose to tentatively annotate the biochemical activity of yeast KRE33 with that of its bacterial ortholog, i.e. the acetylation of RNA.

The situation is different for the second ‘level-1’ candidate with clearly deviating annotations in *E. coli* (AAC75174) and yeast (CFD1; Supplementary Figure S1B). AAC75174 acts as a Na^+^/H^+^ antiporter in the inner membrane and increases the activity of the malate:quinone oxidoreductase (64). CFD1 together with NBP35 forms a cytoplasmic complex that is involved in iron–sulfur protein assembly (65). In the feature architectures this functional difference between the orthologs is reflected by the presence of a transmembrane domain in the bacterial protein, which is not seen in the CFD1. Thus, all evidence points toward a change in the biochemical activity of the soluble domain during evolution of these proteins. Currently, however, we have to leave it open which of the activities, if any, represents that of the ancestral protein and hence have kept this protein in the analysis.

Overall our evaluation reveals that there is a very good chance for ‘level-1’ and ‘level-2’ candidates sharing the same biochemical activity with their corresponding yeast RBF. We therefore applied the same limits to the curation of the two subsets described above. The results for the archaeal and bacterial candidates are shown in Figure 4 and Supplementary Table S6 and for the eukaryotic proteins in Supplementary Table S5. Considering only ‘level-1’ and ‘level-2’ candidates we adjusted the phylogenetic age estimate for 77 RBFs to younger dates. Notably, in 50 of these cases we rejected all archaeal and bacterial orthologs leaving the corresponding RBF confined to the eukaryotes.

### Phylostrata in the ribosome biogenesis pathway of yeast

**Figure 5.**
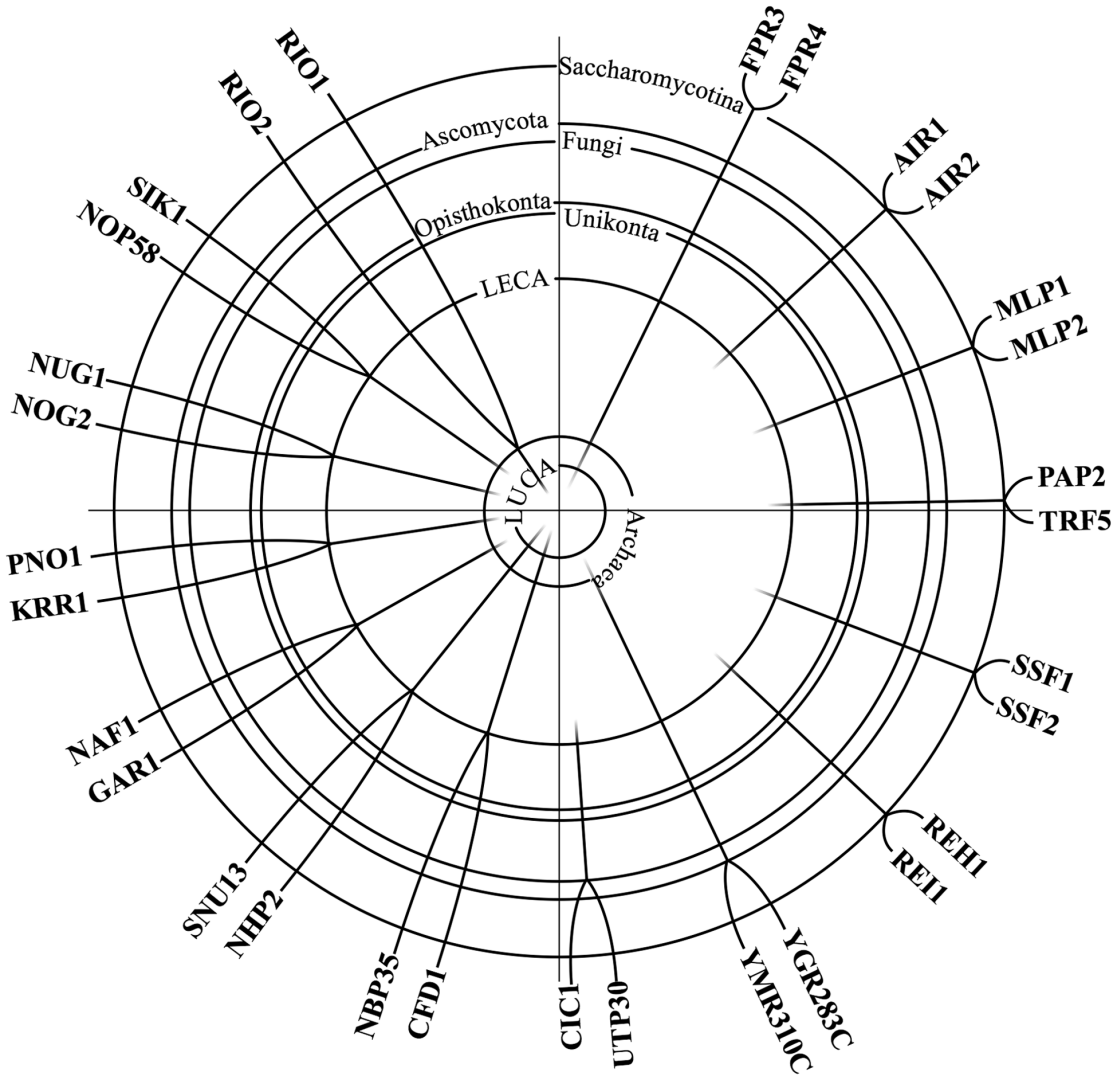
Gene duplication events in the evolution of yeast RBFs. Among the 255 RBFs we could identify 15 homologous pairs that originated by a duplication of an ancestral RBF lineage. Six duplication events were dated to the common ancestors of the Saccharomycotina and eukaryotes, respectively. The remaining three occurred in the common ancestors of the ascomycetes, the fungi, and of the eukaryotes and archaea, respectively. The individual lineages extend to the phylostratum to which the corresponding RBFs can be assigned.

**Figure 6.**
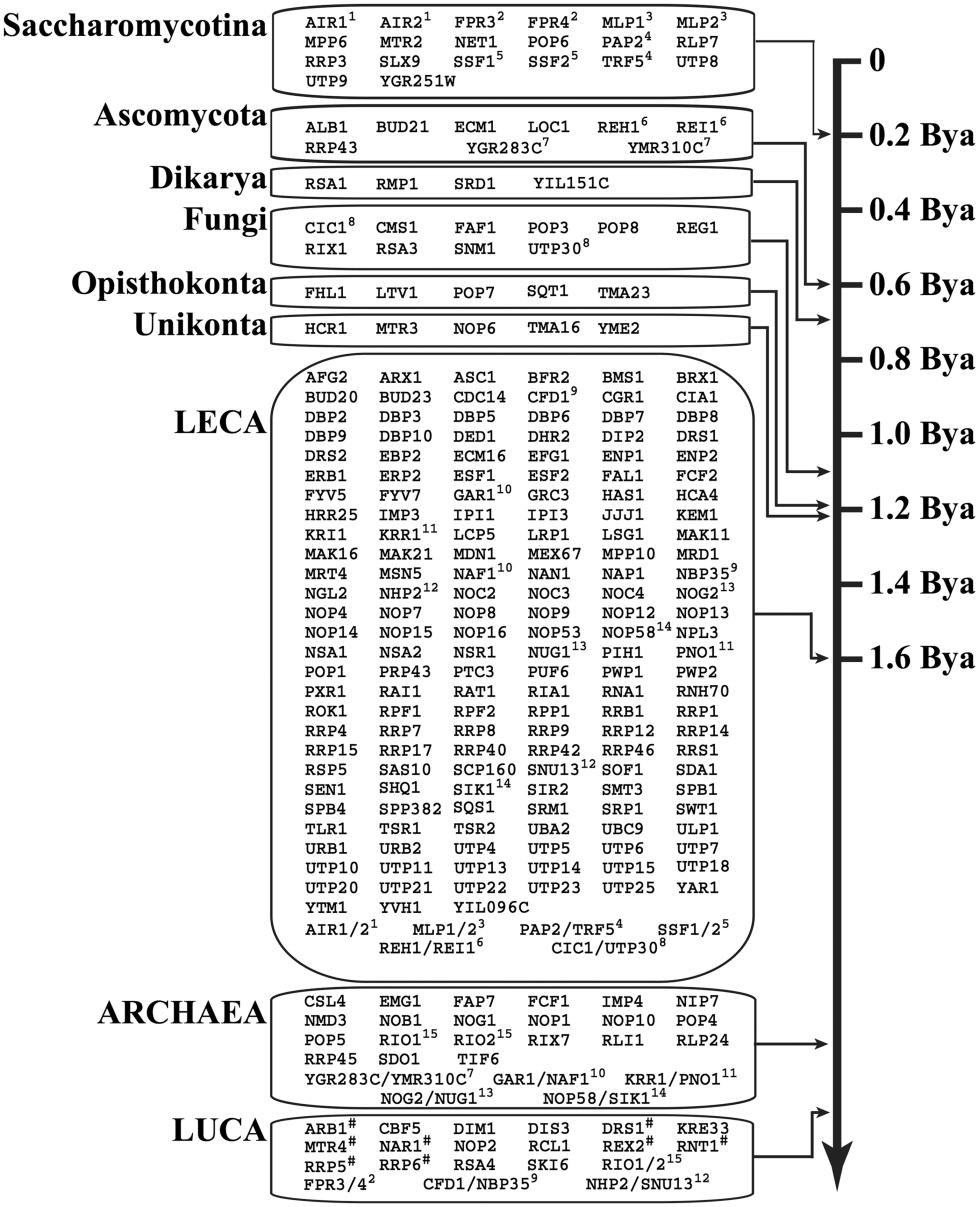
Phylostrata of the yeast ribosome biogenesis pathway. The different strata summarize the minimal evolutionary age estimates for the 255 yeast RBFs. The individual datings integrate the results from the ortholog searches in 344 species, the subsequent analysis of shared functional domains and in part manual curation (see text). The age estimates in billion years are taken from the timetree of life project. LECA, last eukaryotic common ancestor. RBFs marked with a ‘#’ represent possible xenologs present only in bacteria and eukaryotes (see Supplementary Figure S3 for the corresponding phylogenetic tree reconstructions). The 15 pairs of homologous yeast RBFs are each identified by the same superscript numbers. Each pair is represented in two layers. The older layer denotes the minimum age of their shared evolutionary lineage, whereas the younger layer is the one where they have been first identified as separate RBFs (cf. Figure 5).

We have traced 255 yeast proteins with a proposed involvement in ribosome biogenesis throughout the tree of life. We took into account evolutionary relationships of the analyzed species and likely biochemical activities of the identified candidates. The resulting RBF repertoires for the individual species are given in Supplementary Table S8. Before we use this data for compiling the phylostrata in the yeast pathway we have to consider that we, so far, analyzed the yeast RBFs as if they were entirely independently evolving entities. However, those yeast RBFs that originated from a duplication of the same ancestral gene share part of their evolutionary history. Therefore, their evolutionary lineages coalesce within the time over which we can trace them back in evolutionary history. Using a three-step strategy combining orthology inference between yeast and species with increasing evolutionary distance and domain analysis we identified 14 pairs of such homologous yeast RBFs and a 15th pair, RIO1-RIO2, was identified via phylogenetic tree reconstruction (Figure 5; Supplementary Figure S2). Taking these relationships into account, we then subsumed all RBFs of the same age in phylostrata. In each stratum we represented only the evolutionarily distinct RBF lineages. These strata now shed light on the evolution of the yeast pathway (Figure 6).

*Stratum LUCA. *The oldest stratum dates back to the last universal common ancestor (LUCA) shared by all three domains of life and comprises 20 RBF lineages. Fifteen of the corresponding RBFs catalyze an enzymatic reaction (75%; GO-term GO:0003824), whereas only 36% of all 255 proteins are annotated with a catalytic activity in the GO database (66). Moreover, five of the eight RBFs associated with U5 snRNA 3′ end processing term (GO0034476) are found in this oldest stratum. This suggests that the essential and evolutionary conserved key processes in ribosome biogenesis involve a catalytic activity. Alternatively, it is possible that homologs to proteins with a catalytic activity are simply easier to trace over very large evolutionary distances. The question remains, however, whether RBFs in the LUCA layer have been already involved in ribosome biogenesis in the primordial ancestor. The evidence is unambiguous for DIM1 and DRS1, whose counterparts in *E. coli* also participate in ribosome assembly (67,68). Less clear is the situation for the remaining proteins as there is no indication that they contribute to this pathway in *E. coli*. Ribosome assembly is well understood in bacteria and ribosomes can even be assembled *in vitro* (69). This leaves little room for the detection of novel components that have been hitherto overlooked. It rather seems that in these cases an ancient protein whose biochemical activity has remained unchanged throughout the evolution of organismic life was recruited to different pathways on the bacterial and eukaryotic lineages.

For the phylostratum LUCA there is one additional aspect to consider. Some RBF lineages may have been horizontally introduced into the eukaryotes, e.g. via the internalization of an α-proteobacterium by the primordial eukaryote to form present day mitochondria. The presence of such xenologs (70) would compromise our age dating in individual cases as the corresponding RBFs have not necessarily been present in LUCA. To address this issue we computed phylogenetic trees exemplarily for the eight RBF lineages represented only in bacteria and eukaryotes and absent in archaea, which are most suspicious for containing xenologs. In no instance we find in a clear support for a nested placement of the eukaryotes within the bacteria and here particularly within the α-proteobacteria (Supplementary Figure S3). However, an accurate phylogeny inference over these evolutionary distances with individual proteins is hard and the trees are poorly resolved. Thus, we cannot decisively rule out the possibility of a horizontal gene transfer, and thus have marked the corresponding lineages in the LUCA layer.

*Stratum Archaea*.**Stratum *Archaea* (Figure 6) of the yeast ribosome biogenesis pathway dates back to the last common ancestor of the archaea and eukarya. The 26 RBF lineages in this layer represent 31 contemporary yeast RBFs distributed across all stages of ribosome biogenesis. No molecular function GO term is enriched in this set. Taking into account the 12 RBF lineages (16 yeast RBFs) from the stratum LUCA that are found also in the archaea (Figure 4) there is strong evidence for the presence of 38 yeast RBF lineages in contemporary archaea. We will discuss the functional implications of this finding below. Note, that so far our conclusions concerning the stratum *Archaea* depend on the assumption of monophyletic archaea as the third domain of life (33,34). However, recent analyses support an alternative grouping, the *eocyte* hypothesis (71). It is proposed that eukaryotes originated from within the archaea (72,73) and are more closely related to the Crenarchaeota than to the Euryarchaeota. Our results are stable with respect to this alternative phylogeny. All 38 RBF lineages are found either both in Crenarchaeota and the Euryarchaeota, or only in the Euryarchaeota (Figure 4). Thus, our dating of the evolutionary RBF origins is independent from whether or not archaea form a monophylum.

*Eukaryotic strata*.**The bulk of yeast RBFs is found in stratum *LECA* (Figure 6) representing the last eukaryotic common ancestor. This clearly indicates that the main principles of ribosome biogenesis, as it is nowadays seen in yeast, have been laid out already prior to the diversification of contemporary eukaryotes, roughly 1.6 billion years ago (74). Only five genes each are confined to the unikonts, and further five genes are younger than the separation of the Amoebozoa from the common ancestor of fungi and animals (opisthokonts). The remaining four strata comprise 43 genes that are present only in fungi. It is noteworthy that 16 RBFs arose by a duplication of an older RBF lineage (cf. Figure 5). It is among these genes where we can expect to find candidates facilitating fungal-specific adaptation of the ribosome biogenesis pathway. Interestingly, 20 RBFs are younger than the split of the Saccharomycotina from the other ascomycetes roughly 200 million years ago. This suggests that the major part of lineage-specific fine-tuning of ribosome biogenesis in yeast occurred in considerably recent evolutionary times. The RNase MRP complex comprising 10 RBFs (POP1, POP3-8, RMP1, RPP1 and SNM1) serves as one example. Four of these RBFs belong to the group of evolutionarily young factors confined to fungi (POP3, POP6, POP8 and SNM1; Figure 6). Note that a corresponding enzyme complex also exists in humans (21). Thus, it seems that the functionality of the RNase MRP has been adapted in recent fungal evolution.

### Ribosome biogenesis in archaea

**Figure 7.**
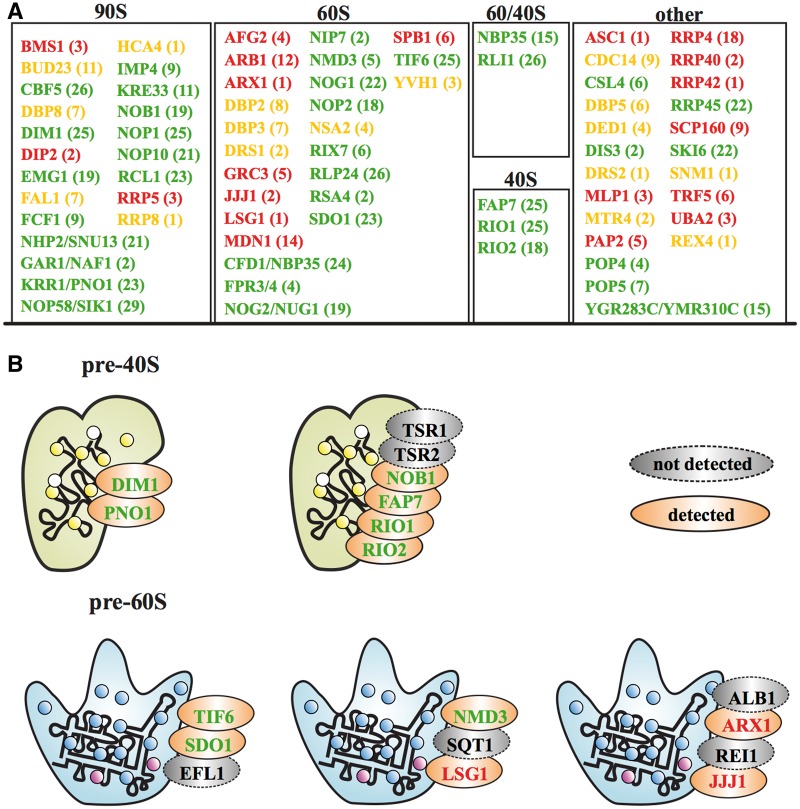
The repertoire of eukaryotic RBFs in archaea. (**A**) A summary of all yeast RBFs with at least some evidence for their presence in the archaea. All candidates have been subjected to manual curation: green, ‘level-1/2’; yellow, ‘level-3’; red, ‘level-4’. The number of species with a detectable ortholog is given in parenthesis. (**B**) The yeast complexes involved in the final processing of the pre-ribosomal complexes in the cytosol are shown together with the involved protein components. Factors without detectable counterparts in the archaea are shown as gray-shaded ellipses. The font color code is as in (A).

Comparative studies have revealed that the archaea take an intermediate position by sharing properties with both the Bacteria and the Eukarya (75,76). Ribosome biogenesis has not been extensively studied in this domain, and little is known about the factors involved in this process (35,36,77). The evolutionary stratigraphy for the yeast ribosome biogenesis pathway indicates that at least 38 RBF lineages are old enough and feature-wise similar enough (curation ‘level-1’ or ‘level-2’) to represent high-confidence candidates contributing to archaean ribosome assembly (Figure 7A). These are distributed over many relevant functional sub-complexes of eukaryotic ribosome biogenesis. Fourteen assemble with the 90S pre-ribosomal complex in yeast, 12 with pre-60S, 2 with both pre-60S and pre-40S and 3 with pre-40S complexes. The remaining seven RBF lineages have been proposed for accessory functions. For the two pre-40S complexes we have detected six of the eight interacting factors as high confidence candidates [Figure 7B; (78)]. For the other three complexes we have found only 3 of the 10 involved RBFs. However, here we face the typical dilemma in candidate searches. Our stringent selection criteria result in reliable candidates with a low false positive rate to the cost of lower sensitivity. Listing all 76 RBF lineages for which we have at least some indication of their presence in archaea (Figure 7A) ameliorates this problem. We can reconstruct the archaean ribosome biogenesis pathway using the high-confidence candidates as scaffold. Gaps in the functional interaction network can then be filled with candidates of lower ranks giving the possibility of their re-evaluation. We have shown this exemplarily in Figure 7B for the cytosolic pre-40S and pre-60S complexes. Our analyses suggest that four of five complexes involved in cytosolic processing in yeast are also present in archaea. In addition to the sub-complexes shown in Figure 7 we found all components of the box C/D snoRNP (NOP1, SIK1, NOP58, SNU13) and of the box H/ACA snoRNP (NOP10, GAR1, CBF5, NHP2). Notably, the latter two RNA–protein complexes have already been functionally and structurally characterized in archaea [reviewed in (2)] highlighting the significance of our predictions.

### Evolutionary plasticity in eukaryote ribosome biogenesis

One of the prominent findings of our study is the high evolutionary age of most of the analyzed yeast genes. The evolutionary ancestry for 80% of the RBFs reaches back at least to the common ancestor of all eukaryotes (Figure 6). At first sight this suggests that this pathway has with some exceptions remained almost invariant throughout evolution, and innovations are limited to the addition and removal of a small number of factors. This notion seems further supported by the similarity in the (pre-)rRNA processing pathways of yeast, human and *Arabidopsis* (32,79). However, already now there is also evidence for differences in the pathway between species, such as variations in the processing pathway of the rRNAs (13,80) and in the set of co-factors involved. DOMINO1, for example, is a gene specific to plants that is presumably involved in ribosome biogenesis (81). Similarly, the nuclear export receptor Exportin 5 is of importance for human ribosome biogenesis, while its ortholog in yeast has not been implicated in this pathway so far (23). Indeed, when we take into account the representation of the individual factors within the supertaxa (Figure 2) we see room for plasticity. As an example, orthologs to 233 yeast factors were found in at least one of the 94 animal species (Figure 2A, ‘ALL’). This number reduces to 211 when we count only candidates seen in at least 25% of the species, and 197 are represented in at least half of the species. Although the fractions of RBFs identified might not be complete—a consequence of the draft status of many analyzed genomes—the extent of missed orthologs to yeast RBFs is most likely moderate. The genomes of the three model species human, mouse and drosophila are considered finished and should be reasonably well annotated. Within the corresponding annotated gene sets we find orthologs to almost the same—yet surprisingly low—number of yeast factors (human: 200, mouse: 197, drosophila: 196). However, the factor sets are not identical. Only 181 factors are found in all three species, while the union of the three sets comprises 212 factors. This simple example has two relevant implications. It shows that a comprehensive taxon sampling is essential for tracing the evolutionary history of yeast RBFs. Furthermore, it demonstrates that lineage-specific losses of individual RBFs are characteristic for the recent evolution of ribosome biogenesis.

### Ribosome biogenesis factors from a human perspective

Up to this point our study was limited to assessing the loss of yeast RBFs when traversing the tree of life toward taxa with increasing evolutionary distance. What remains unexplored is the fraction of factors that have been added to this pathway in species other than yeast, but also which ancestral RBFs have been lost in yeast. A complementary analysis based on a comparable collection of RBFs from a different model organism outside the fungi could provide valuable information for addressing these points. The available data for such an analysis are scarce, yet an RNAi-based screen for factors potentially involved in human ribosome biogenesis can serve as a start (23). Note however, that also this screen was mainly focusing on putative homologs of yeast RBFs and considered only a small number of other candidate cofactors (e.g. nuclear transport receptors). The resulting candidate list is therefore tentative and does not represent an unbiased genome-wide screen. Still the extent of overlap to the human proteins identified by our phylogenetic profiling procedure gives at least some indication about the level of innovation in the human pathway relative to that of yeast. Of the 153 factors identified by (23) we excluded the 61 ribosomal proteins as they were not part of our analysis. Of the remaining 92 human factors 62 have been identified also in our analysis as human RBF candidates (Supplementary Table S9). This indicating an overall good agreement between the two studies and lends independent support to our results. One RBF (RBM23) was missed as we could not reproduce the proposed orthology to yeast NOP13 in our set, and two further candidates of Wild *et al.* (2010) do not possess an ortholog in yeast. The remaining 27 human factors are interesting as there is so far no indication that their corresponding yeast orthologs are associated with ribosome biogenesis. If these proteins are truly involved in human ribosome biogenesis, they represent the first major step toward unraveling the differences in ribosome biogenesis between individual eukaryotic species.

## CONCLUSION

Ribosome biogenesis as it is nowadays seen in *S. cerevisiae* is in major parts an evolutionarily ancient process. The emergence of most of the involved proteins date back to the root of the eukaryotic phylogeny, and there is good indication that 20 RBF lineages existed already in the LUCA. What fraction of these oldest factors has been involved in primordial ribosome assembly remains to be explored. Although we could identify *E. coli* counterparts with comparable biochemical activity for 12 yeast RBFs, only 2 are known to participate in bacterial ribosome assembly. This indicates that the recruitment of individual proteins to this pathway has been largely independent in the bacterial and eukaryotic lineages. The situation seems different for archaea, the sister domain of the eukaryotes. We provide evidence that several functional sub-complexes of yeast ribosome biogenesis exist in the archaea as well. Among these are cytosolic pre-60S and pre-40S complexes as well as the box C/D snoRNP and box H/ACA snoRNP. Our findings are in line with previous experimental characterizations of the latter two complexes in archaea, and thus our archaean RBF repository forms a solid basis for studying ribosome biosynthesis in this domain. Within the eukaryotes the general layout of the pathway seems by and large stable as the majority of yeast RBFs are found throughout the eukaryotic tree of life. However, there is also evidence for lineage-specific plasticity. About 15% of the yeast RBFs appear confined to the fungi indicating that the corresponding proteins are evolutionarily young. For the remaining proteins there is strong evidence for independent and lineage-specific losses, as shown on the examples of humans, mouse and drosophila. Shifting the focus toward an initial pan-species comparison of RBF pathways provides further evidence for lineage-specific differences. For two alleged human RBFs we find no orthologs in yeast. Additional 27 human RBF candidates possess yeast orthologs, which however appear not associated with ribosome biogenesis in yeast. Future analyses on the plasticity of ribosome biogenesis for a given species will have to concentrate on the identification of such lost or newly gained RBFs. It will be particularly interesting to investigate to what extent duplications and subsequent diversifications of established RBF-encoding genes drive the more recent evolution of the ribosome biogenesis pathway.

## SUPPLEMENTARY DATA

Supplementary Data are available at NAR Online.

Supplementary Data
