# Supplementary material for: The evolution of the ribosome biogenesis pathway from a yeast perspective
Source: Nucleic Acids Res. 2013 Nov 14;42(3):1509–23. doi: 10.1093/nar/gkt1137 (PMC3919561; doi:10.1093/nar/gkt1137)
Supplement: Supplementary Data [file supp_42_3_1509__index.html]

The evolution of the ribosome biogenesis pathway from a yeast perspective — The evolution of the ribosome biogenesis pathway from a yeast perspective — Supplementary Data 

# The evolution of the ribosome biogenesis pathway from a yeast perspective

## Supplementary Data

files

**Files in this Data Supplement:**

- Supplementary Data - pdf file
- Supplementary Data - pdf file
- Supplementary Data - xlsx file
- Supplementary Data - xlsx file
