# Supplementary material for: The evolution of the ribosome biogenesis pathway from a yeast perspective
Source: Nucleic Acids Res. 2013 Nov 14;42(3):1509–23. doi: 10.1093/nar/gkt1137 (PMC3919561; doi:10.1093/nar/gkt1137)

Supplementary Figure 1: Analysis of the *E. coli* orthologs of KRE33 and CFD1.
(**A**) We used the bacterial proteins AAC75527 (ortholog of KRE33) as query for a FACT search and a Blast search against all proteins annotated in the genome of *Saccharomyces cerevisiae*. The FACT search identifies KRE33 as the best scoring protein and there is no other yeast protein achieving a comparable score. The BLAST search reveals that among all yeast proteins only KRE33 (YNL132W) shares a significant sequence similarity (e-value: 1e-15) with the bacterial protein. The overall good agreement of the feature architectures both with respect to order and length is shown in the feature dotplot (generated with the online tool at http://fact.cibiv.univie.ac.at/CompareResult_HMM.php). (**B**) shows the same analysis for the bacterial protein AAC75174, the *E. coli* ortholog to yeast CFD1.

A

Top 3 FACT results:

query id	hit id	MS_uni	MS_st	FACT	Lin
AAC75527	YNL132W	0.553	0.933	0.743	0.605
AAC75527	YFL017C	0.312	0.054	0.243	0.509
AAC75527	YDR071C1	0.279	0.054	0.242	0.495

BLAST result:

query id	hit id	bit score	e-value	MS_uni	MS_st	FACT	Lin
AAC75527	YNL132W	79.7	1e-15	0.553	0.933	0.743	0.605
AAC75527	YDR095C	27.7	6.2	0.084	0.00	0.00	0.403
AAC75527	YOR227W	27.3	8.0	0.234	0.00	0.00	0.511

Feature Dot Plot

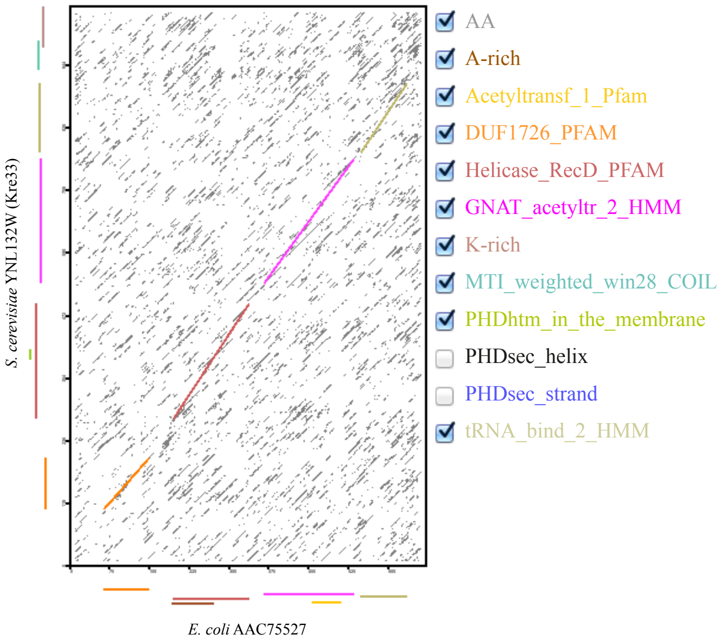

B

Top 5 FACT results:

query id	hit id	MS_uni	MS_st	FACT	Lin
AAC75174	YGL091C	0.634	0.593	0.670	0.727
AAC75174	YIL003W	0.622	0.593	0.605	0.714
AAC75174	YDL100C	0.521	0.593	0.582	0.670
AAC75174	YPR088C	0.255	0.051	0.239	0.428
AAC75174	YJL194W	0.267	0.000	0.200	0.466

Blast result:

query id	hit id	bit score	e-value	MS_uni	MS_st	FACT	Lin
AAC75174	YIL003W	155	1e-38	0.622	0.593	0.605	0.714
AAC75174	YGL091C	140	1e-34	0.634	0.593	0.670	0.727
AAC75174	YBR150C	26.9	5.2	0.088	0.00	0.00	0.351
AAC75174	YML076C	26.9	5.2	0.147	0.00	0.00	0.437
AAC75174	YPL240C	26.6	6.7	0.120	0.00	0.00	0.398

Feature Dot Plot

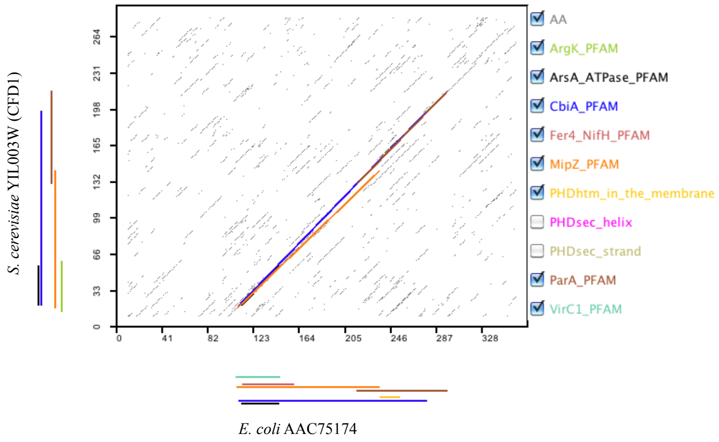

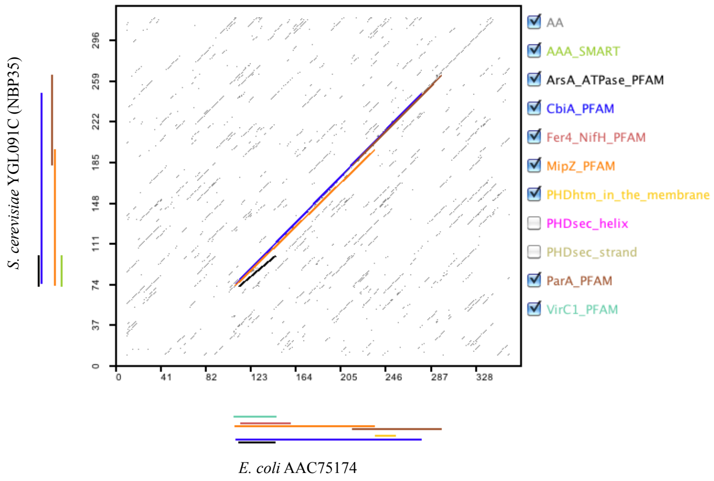

Supplementary Figure 2. The evolutionary relationships of RIO1 and RIO2
Maximum likelihood tree reconstruction reveals that RIO1 and RIO2 are ancient paralogs that can be traced back to a gene duplication that occurred in the last common ancestor of eukaryotes and archaea. Details on the tree reconstruction procedure are given in the main text.

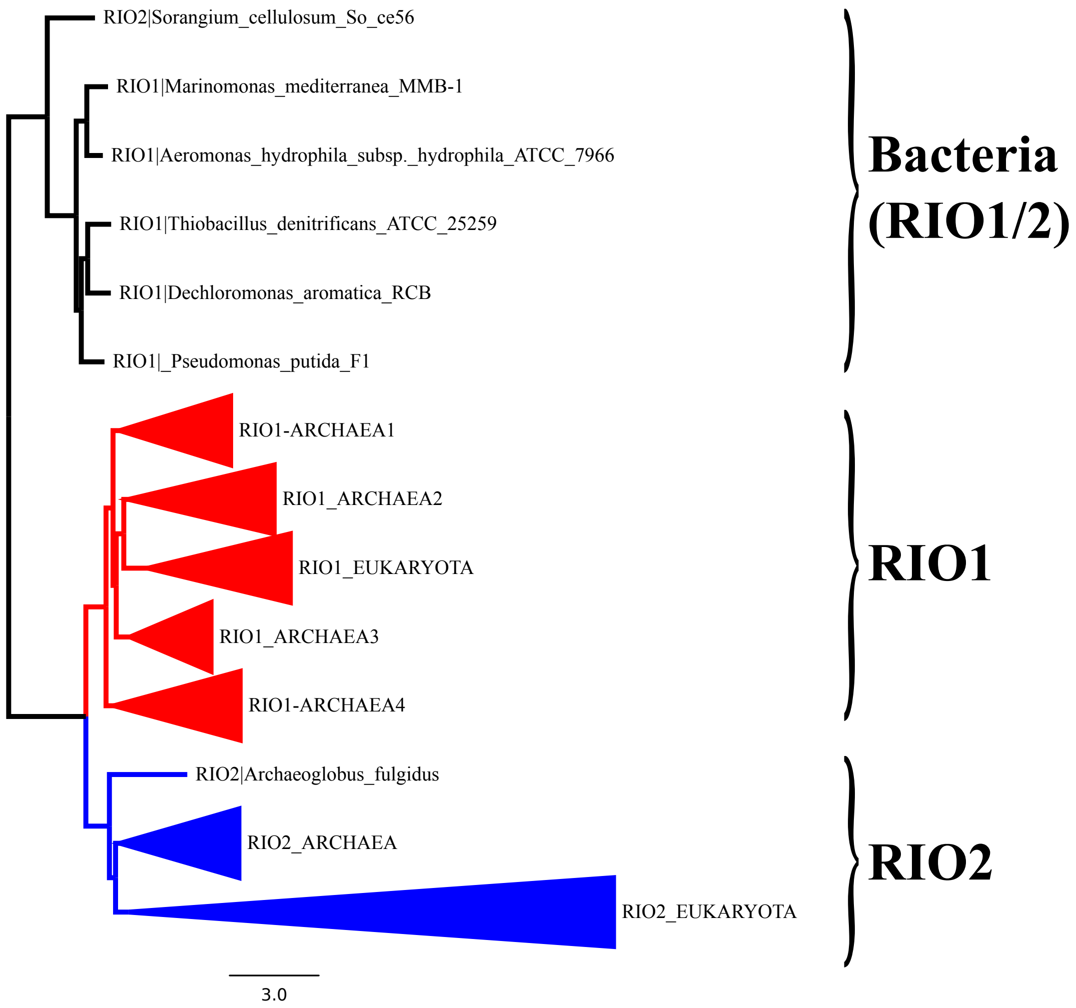

Supplementary Figure 3: Maximum likelihood tree reconstruction for eight RBF lineages present only in bacteria and eukaryotes.

We computed maximum likelihood gene trees to assess the possible presence of xenologs. In brief, the orthologous sequences were aligned with mafft (L-ins-I), alignment columns with more than 50% gaps where removed, and maximum likelihood trees were computed with RAxML v 7.3 (PROTGAMMAILGF) performing 100 bootstrap replicates. We find in no instance a clear support for a nested placement of the eukaryotes within the bacteria and here particularly within the alpha-proteobacteria, the alleged closest relatives of the mitochondria.

Supplementary Figure 3A: ML tree of ARB1

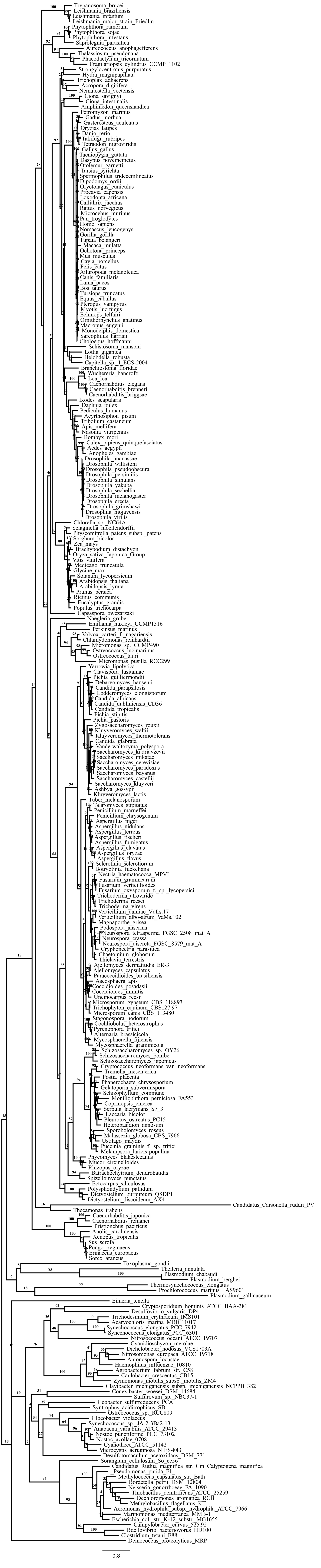

Supplementary Figure 3B. ML tree of DRS1

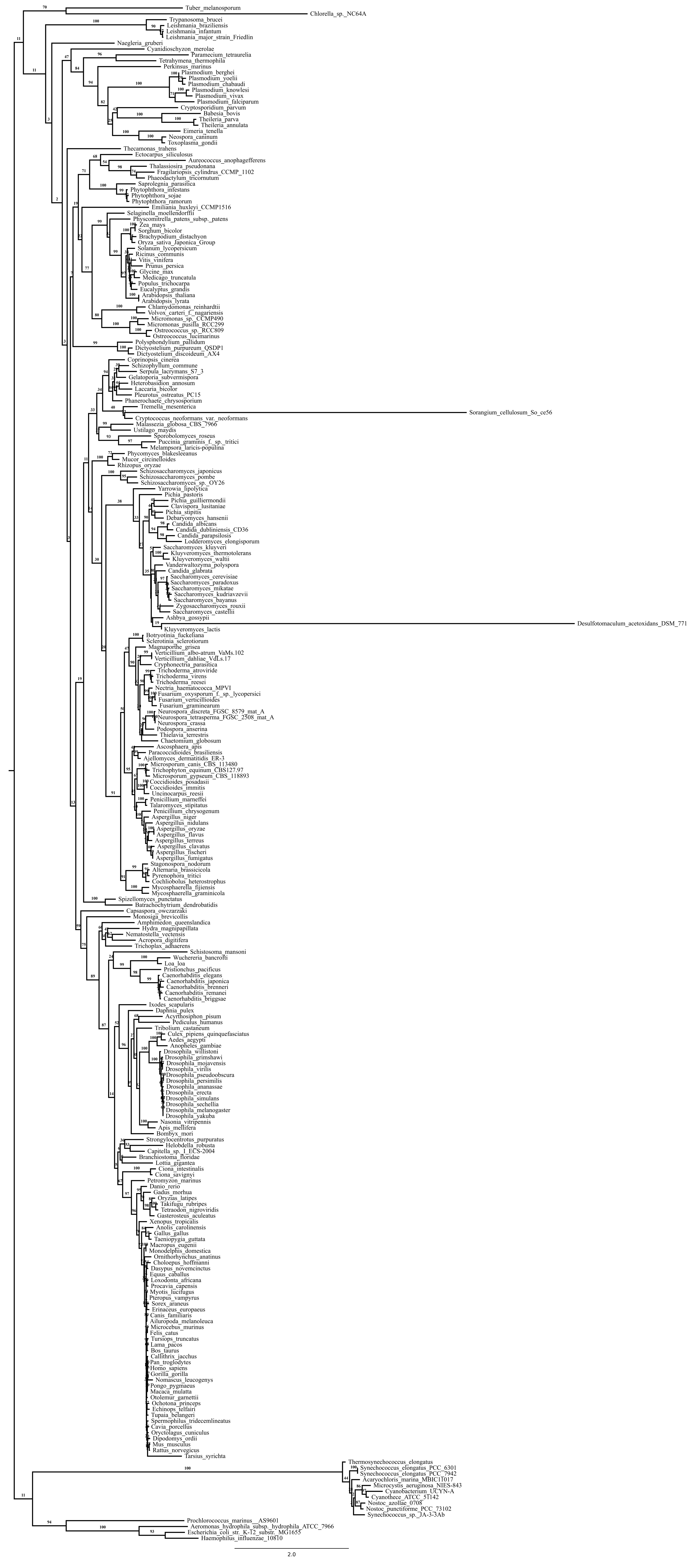

Supplementary Figure 3C. ML tree of MTR4

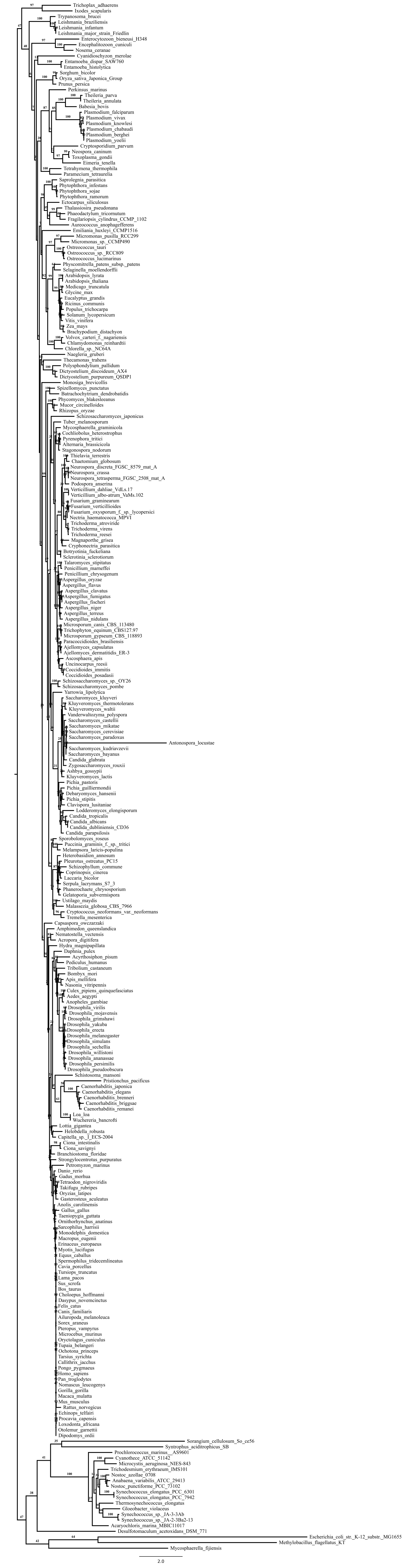

Supplementary Figure 3D. ML tree of NAR1

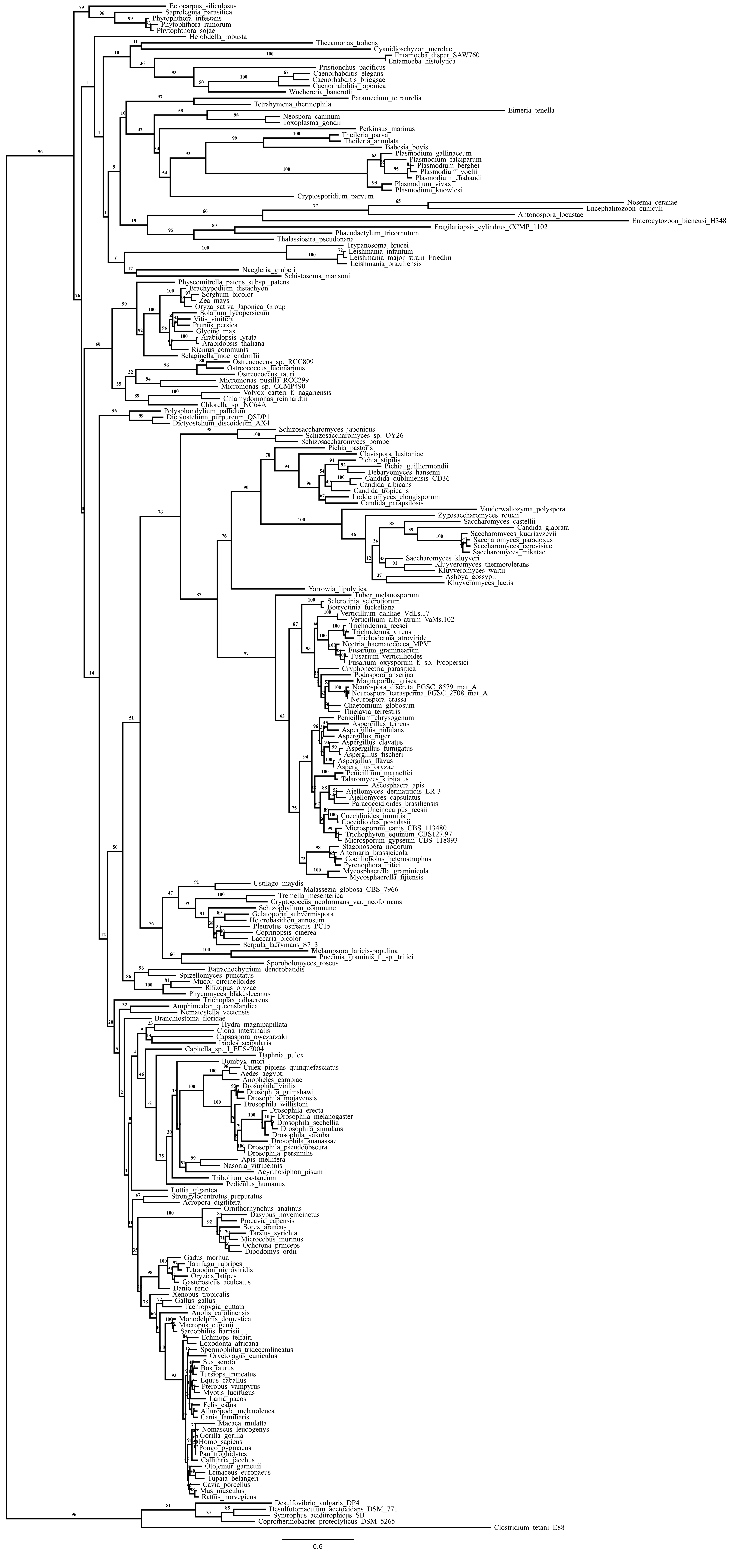

Supplementary Figure 3 E. ML tree of REX2

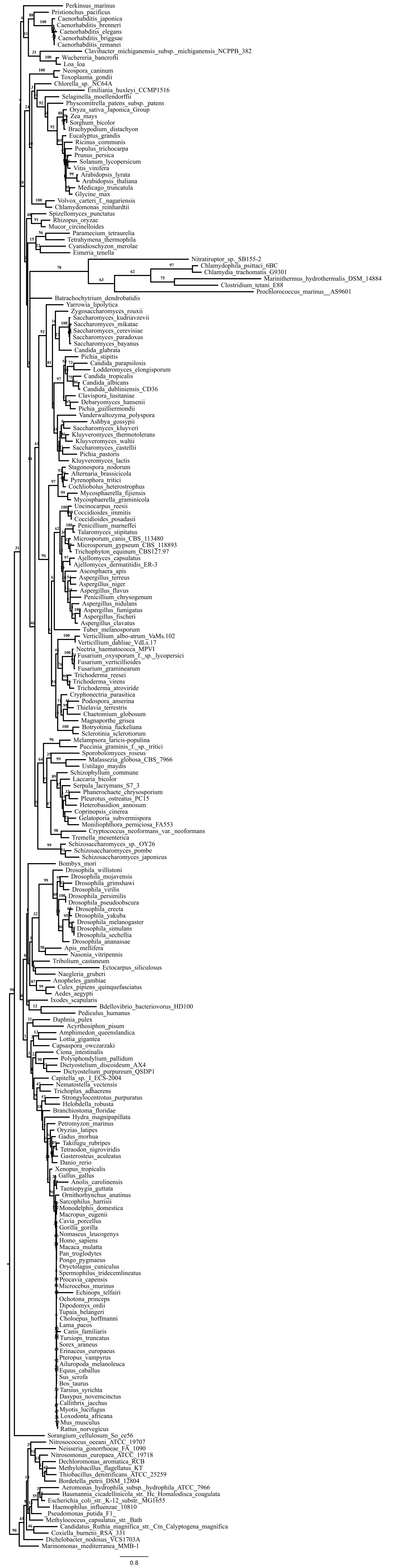

Supplementary Figure 3 F. ML tree of RNT1

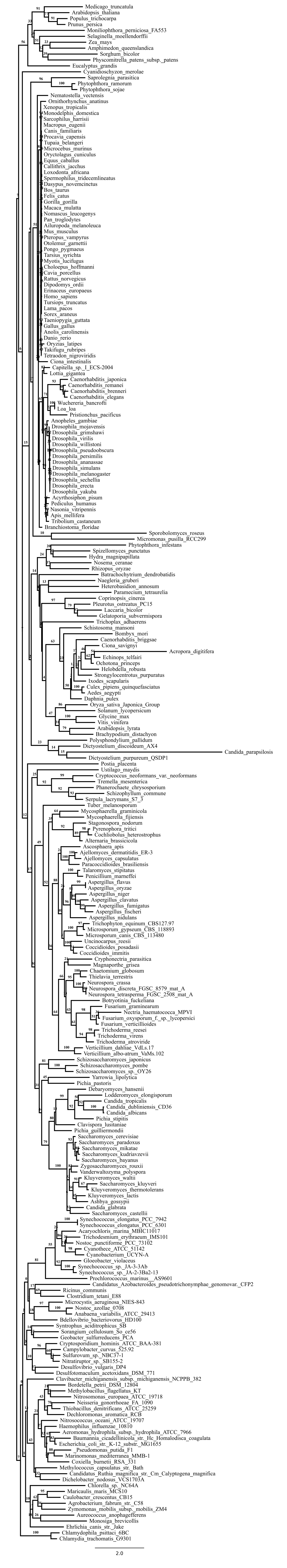

Supplementary Figure 3 G. ML tree of RRP5

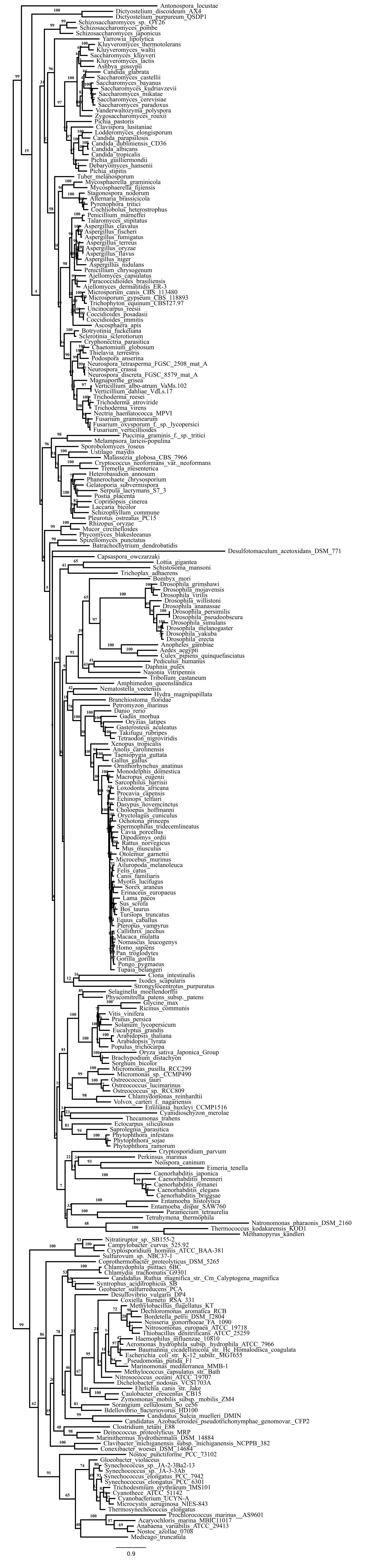

Supplementary Figure 3 H. ML tree of RRP6

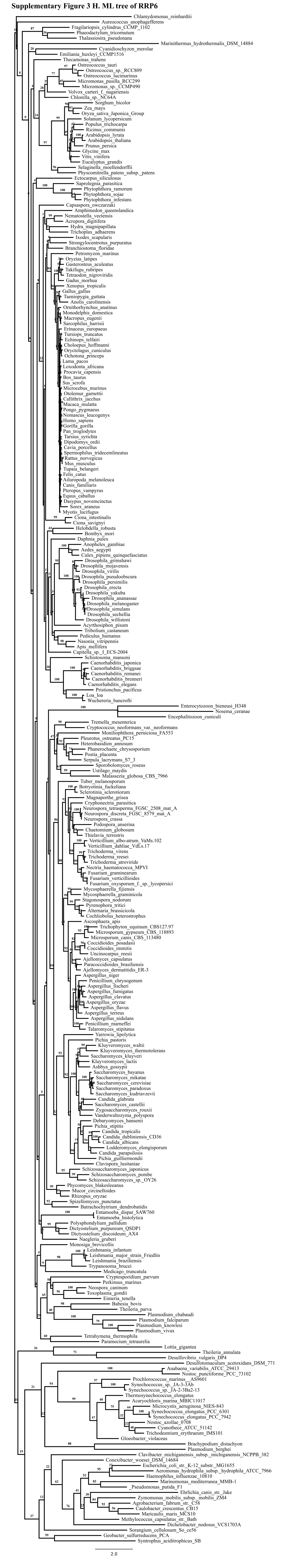

Supplement: Supplementary Data [file supp_gkt1137_nar-02229-n-2013-File009.pdf]
