# Supplementary material for: The evolution of the ribosome biogenesis pathway from a yeast perspective
Source: Nucleic Acids Res. 2013 Nov 14;42(3):1509–23. doi: 10.1093/nar/gkt1137 (PMC3919561; doi:10.1093/nar/gkt1137)
Supplement: Supplementary Data [file supp_gkt1137_nar-02229-n-2013-File010.pdf]

Supplementary Text 1. Refined search for yeast RBFs in *E. cuniculi*.

In our analysis so far we could identify counterparts only to 127 yeast RBFs in the Microsporidia. This is among all analyzed eukaryotes by far the lowest number despite the alleged close relationships of fungi and Microsporidia (1), and despite the fact that we have analyzed 4 different microsporidian species. However, our screen for the presence of counterparts to the 255 yeast RBFs in individual taxa is based on the identification of orthologs. Obviously, orthology inference will fail in instances where a protein, although present, has changed to an extent that it no longer suffices to induce a reciprocal best (Blast) hit. Even worse, sequence similarity may not even suffice to trigger a significant Blast hit at all. It is easy to imagine that particularly species with an increased evolutionary rate, such as the Microsporidia (e.g. (2)), will suffer from the resulting underestimation of the number of RBFs.

To assess the likely impact of this aspect we performed a refined search for the 128 undetected RBFs in the gene set of *Encephalitozoon cuniculi*. To increase the sensitivity we used a unidirectional Blast (3) search for *E. cuniculi* proteins with a significant sequence similarity to a yeast RBF, and a FACT search (4) to detect proteins with a similar feature architecture to a yeast RBF. The complementarity of both approaches in the search for proteins with comparable biochemical activity has been demonstrated before (4).

Subsequently, the results of both searches were integrated and manually curated. In brief, we considered all Blast hits below an e-value of 1E-10. This list was complemented with all highest scoring *E. cuniculi* proteins from the FACT search where the corresponding feature architecture appeared unique in *E. cuniculi*. The resulting list comprised 29 distinct candidates, 13 of which did not meet the e-value threshold in the Blast search (Table 1). All initial candidates served then as a query for a reverse search in yeast via a WU-BlastP hosted at the Saccharomyces Genome Database (http://www.yeastgenome.org/cgi-bin/blast-sgd.pl) and via FACT (Table 1). This revealed for most proteins either a substantially higher sequence similarity to a non-RBF or multiple comparable Blast hits paired with a feature architecture occurring several times in yeast. In six cases, however, we detected promising RBF candidates in *E. cuniculi* that have been overlooked in our orthology-based approach. Notably, 4 of these could be identified only via similarity of their feature architectures (CSL4, ENP1, POP4, and RRP1) as they did not trigger a significant Blast hit.

In summary, this refined analysis shows that, as expected, our orthology-based search in the fast evolving Microsporidia has overlooked six promising RBF candidates. Notably, four of these new candidates lack a significant sequence similarity to any yeast protein demonstrating the sensitivity of our refined search. Still, we found evidence for only six of the 128 absent yeast RBFs indicating that the vast majority of the remaining 122 RBFs truly have been lost in the Microsporidia.

Yeast RBF	*E. cuniculi* candidate*	Evidence	Bit Score (e-value)	Curation
AFG2	Q8SSJ5	F/B	461 (1E-131)	CDC48 (3E-231)
AIR1	Q8SU59	B	59 (4E-10)	AIR1/AIR2 (3E-12)
CDC14	Q8SS66	F	33 (0.04)	Feature architecture occurs multiple times in yeast
CSL4	Q8SVT7	F	24 (9.9)	CSL4
DBP6	Q8SR63	F/B	120 (1E-28)	RRP3 (2E-71)
DBP7	Q8SR63	F/B	157 (1E-39)	RRP3 (2E-71)
DBP8	Q8SR63	F/B	243 (1E-65)	RRP3 (2E-71)
DED1	Q8SRB2	F/B	242 (3E-65)	DBP2 (1.4E-122)
DRS1	Q8SR63	F/B	224 (9E-60)	RRP3 (2E-71)
ECM16	Q8SR50	F/B	164 (1E-41)	ECM16 (1E-75); similar hits exist among the PRP family)
ENP1	Q8STP8	F	-	ENP1 (reciprocal best FACT hit)
ERP2	Q8SWC3/Q8SS84	F	-	Feature architecture occurs multiple times in yeast
FHL1	Q8SQU6	B	84 (2E-17)	FKH2 (2.7E-20)
MAK11	Q8SRB0	F	-	ASC1 (3.3E-15) FA occurs multiple times in yeast
MAK11	Q8SSJ2	B	65 (7E-12)	COP1 (4E-74)
MAK5	Q8SRB2	F/B	150 (1E-37)	DBP2 (1.4E-122)
MLP1	Q8SWN0	F/B	67 (6E-12)	Blast/FA unspecific mutiple hits
MLP2	Q8SS35	B	100 (4E-22)	MYO1 (2.8E-170)
NAP1	Q8SQI8	F	-	Feature architecture occurs multiple times in yeast
NGL2	Q8SU52	F/B	62 (6E-11)	CCR4 (2.6E-52)
NOP13	Q8SSA1	F/B	68 (7E-13)	several equally good FACT/Blast hits
NOP9	Q8SQJ0	F	31 (0.13)	PUF3 (6E-43)
POP4	Q8SUV2	F	-	POP4 (reciprocal best FACT hit)
REX4	Q8SRL4	F	59 (2E-10)	RNH70 (3E-25)
RLP7	Q8SS93	F/B	63 (1E-11)	RPL7 (1E-29)
RRP1	Q8SV05	F	-	RRP1 (reciprocal best FACT hit)
RRP9	Q8SQS4	B	62 (6E-11)	TAF5 (2.3E-66)
RRP42	Q8STZ3	F	-	Feature architecture occurs multiple times in yeast
SEN1	Q8SR02	B	156 (9E-39)	NAM7 (3.5E-128)
SIR2	Q8SSB6	F/B	92 (4E-20)	HST4 (1.8E-31)
SRD1	Q8SSI3	F	32 (0.015)	Feature architecture occurs multiple times in yeast
YAR1	Q8SR46	F	26 (0.9)	Feature architecture occurs multiple times in yeast
YTM1	Q8SRB0	F	49 (3E-7)	ASC1 (3.3E-15) FA occurs multiple times in yeast
YVH1	Q8SS66	F	-	Feature architecture occurs multiple times in yeast

Table 1. Result of the refined search for yeast RBFs in *E. cuniculi*. Evidence: **F**(act) and/or **B**last. Curation gives the result of the reverse search in the yeast protein set using the *E. cuniculi* candidate as query. E-values of the reverse Blast are given when available and when they led to the exclusion/acceptance of a candidate. Color code of the candidate curation follows the main text: Green: *level-1*; light green: *level-2*; yellow: *level-three*; red: *level-4* candidate.

References

1. Corradi, N. and Keeling, P.J. (2009) Microsporidia: a journey through radical taxonomical revisions. *Fungal Biology Reviews*, **23**, 1-8.
2. Brinkmann, H., van der Giezen, M., Zhou, Y., de Raucourt, G.P. and Philippe, H. (2005) An Empirical Assessment of Long-Branch Attraction Artefacts in Deep Eukaryotic Phylogenomics. *Syst Biol*, **54**, 743-757.
3. Altschul, S.F., Madden, T.L., Schäffer, A.A., Zhang, J., Zhang, Z., Miller, W. and Lipman, D.J. (1997) Gapped BLAST and PSI-BLAST: a new generation of protein database search programs. *Nucleic Acids Res*, **25**, 3389-3402.
4. Koestler, T., Haeseler, A.v. and Ebersberger, I. (2010) FACT: Functional annotation transfer between proteins with similar feature architectures. *BMC Bioinformatics*, **11**, 417.

Supplementary Text 2. Comparison of the functional annotations of yeast RBFs and the orthologs in *Escherichia coli*

ARB1

>ARB1|Escherichia_coli_str._K-12_substr._MG1655|AAC73881.2
MNDAVITLNGLEKRFPGMDKPAVAPLDCTIHAGYVTGLVGPDGAGKTTLMRMLAGLLKPDSGSATVIGFDP
IKNDGALHAVLGYMPQKFGLYEDLTVMENLNLYADLRSVTGEARKQTFARLLEFTSLGPFTGRLAGKLSGG
MKQKLGLACTLVGEPKVLLLDEPGVGVDPISRRELWQMVHELAGEGMLILWSTSYLDEAEQCRDVLLMNEG
ELLYQGEPKALTQTMAGRSFLMTSPHEGNRKLLQRALKLPQVSDGMIQGKSVRLILKKEATPDDIRHADGM
PEININETTPRFEDAFIDLLGGAGTSESPLGAILHTVEGTPGETVIEAKELTKKFGDFAATDHVNFAVKRG
EIFGLLGPNGAGKSTTFKMMCGLLVPTSGQALVLGMDLKESSGKARQHLGYMAQKFSLYGNLTVEQNLRFF
SGVYGLRGRAQNEKISRMSEAFGLKSIASHATDELPLGFKQRLALACSLMHEPDILFLDEPTSGVDPLTRR
EFWLHINSMVEKGVTVMVTTHFMDEAEYCDRIGLVYRGKLIASGTPDDLKAQSANDEQPDPTMEQAFIQLI
HDWDKEHSNE

ARB1 is annotated as YbhF in E.coli, which is assigned as ATP-binding component of a predicted ATP-dependent efflux pump, but literature does not exist for the E.coli protein (http://biocyc.org/ECOLI/NEW-IMAGE?type=ENZYME&object=YBHF-MONOMER). Yeast ARB1 is similary annotated. Dong J, et al. (2005) The novel ATP-binding cassette protein ARB1 is a shuttling factor that stimulates 40S and 60S ribosome biogenesis. Mol Cell Biol 25(22):9859-73

578 aa (bacteria) vs. 610 aa (yeast)

BUD23

>BUD23|Escherichia_coli_str._K-12_substr._MG1655|AAC73864.1
MATVNKQAIAAAFGRAAAHYEQHADLQRQSADALLAMLPQRKYTHVLDAGCGPGWMSRHWRERHAQVTALD
LSPPMLVQARQKDAADHYLAGDIESLPLATATFDLAWSNLAVQWCGNLSTALRELYRVVRPKGVVAFTTLV
QGSLPELHQAWQAVDERPHANRFLPPDEIEQSLNGVHYQHHIQPITLWFDDALSAMRSLKGIGATHLHEGR
DPRILTRSQLQRLQLAWPQQQGRYPLTYHLFLGVIARE

BUD23 is annotated as SAM-dependent malonyl-CoA methyltransferase as for example described in Lin S, Hanson RE, Cronan JE. Biotin synthesis begins by hijacking the fatty acid synthetic pathway. Nat Chem Biol. 2010 Sep;6(9):682-8. In contrast, yeast Bud23 is a methyltransferase, which methylates residue G1575 of 18S rRNA required for rRNA processing and nuclear export of 40S ribosomal subunits independently of methylation activity.

251 aa (bacteria) vs. 275 aa (yeast)

CDC14

>CDC14|Escherichia_coli_str._K-12_substr._MG1655|AAC74493.1
MLQGAGWLLLLAPFFFFTYGSLNQFTAVQDLNSHDIPSQVFGWETAIPFLPWTIVPYWSLDLLYGFSLFVC
STTFEQRRLVHRLILATVMACCGFLLYPLKFSFIRPEVSGVTGWLFSQLELFDLPYNQSPSLHIILCWLLW
RHFRQHLAERWRKVCGGWFLLIAISTLTTWQHHFIDVITGLAVGMLIDWMVPVDRRWNYQKPDQRRIKIAL
PYVVGAGSCIVLMELMMMIQLWWSVWLCWPVLSLLIIGRGYGGLGAITTGKDSQGKLPPAVYWLTLPCRIG
MWLSMRWFCRRLEPVSKMTAGVYLGAFPRHIPAQNAVLDVTFEFPRGRATKDRLYFCVPMLDLVVPEEGEL
RQAVAMLETLREEQGSVLVHCALGLSRSALVVAAWLLCYGHCKTVNEAISYIRARRPQIVLTDEHKAMLRL
WENR

Yeast CDC14 is a phosphatase, while nothing is known about the E.coli protein YnbD. However, it is predicted as inner membrane phosphatase (http://biocyc.org/ECOLI/NEW-IMAGE?type=ENZYME&object=G6730-MONOMER).

430 aa (bacteria) vs. 551 aa (yeast)

CFD1
```
>CFD1|Escherichia_coli_str._K-12_substr._MG1655|AAC75174.2
MNEQSQAKSPEALRAMVAGTLANFQHPTLKHNLTTLKALHHVAWMDDTLHVELVMPFVWHSAFEELKEQCS
AELLRITGAKAIDWKLSHNIATLKRVKNQPGINGVKNIIAVSSGKGGVGKSSTAVNLALALAAEGAKVGIL
DADIYGPSIPTMLGAENQRPTSPDGTHMAPIMSHGLATNSIGYLVTDDNAMVWRGPMASKALMQMLQETLW
PDLDYLVLDMPPGTGDIQLTLAQNIPVTGAVVVTTPQDIALIDAKKGIVMFEKVEVPVLGIVENMSVHICS
NCGHHEPIFGTGGAEKLAEKYHTQLLGQMPLHISLREDLDKGTPTVISRPESEFTAIYRQLADRVAAQLYW
QGEVIPGEISFRAV
```

CFD1 In Yeast is a highly conserved, iron-sulfur cluster binding protein localized in the cytoplasm, which forms a complex with Nbp35p that is involved in iron-sulfur protein assembly in the cytosol. In E. coli MRP acts as a Na+/H+ antiporter and increases the activity of the malate:quinone oxidoreductase (Swartz et al. (2006) The Mrp Na+/H+ antiporter increases the activity of the malate:quinone oxidoreductase of an Escherichia coli respiratory mutant. J Bacteriol. 2005 Jan;187(1):388-91. A mutant of the bacterial MRP or ApbC protein in Salmonella typhimurium exhibits a defect in the alternative pyrimidine biosynthetic pathway, and AbpC is proposed to act in formation of 4-amino-5-hydroxymethyl-2-methyl pyrimidine from aminoimidazole ribonucleotide during thiamine biosynthesis in Salmonella typhimurium (Petersen96: Petersen L, Downs DM (1996). "Mutations in apbC (mrp) prevent function of the alternative pyrimidine biosynthetic pathway in Salmonella typhimurium." J Bacteriol 178(19);5676-82. PMID: 8824612)

369 aa (bacteria) vs. 293 aa (yeast)

DBP5
```
>DBP5|Escherichia_coli_str._K-12_substr._MG1655|AAC73866.1
MSKPFKLNSAFKPSGDQPEAIRRLEEGLEDGLAHQTLLGVTGSGKTFTIANVIADLQRPTMVLAPNKTLAA
QLYGEMKEFFPENAVEYFVSYYDYYQPEAYVPSSDTFIEKDASVNEHIEQMRLSATKAMLERRDVVVVASV
SAIYGLGDPDLYLKMMLHLTVGMIIDQRAILRRLAELQYARNDQAFQRGTFRVRGEVIDIFPAESDDIALR
VELFDEEVERLSLFDPLTGQIVSTIPRFTIYPKTHYVTPRERIVQAMEEIKEELAARRKVLLENNKLLEEQ
RLTQRTQFDLEMMNELGYCSGIENYSRFLSGRGPGEPPPTLFDYLPADGLLVVDESHVTIPQIGGMYRGDR
ARKETLVEYGFRLPSALDNRPLKFEEFEALAPQTIYVSATPGNYELEKSGGDVVDQVVRPTGLLDPIIEVR
PVATQVDDLLSEIRQRAAINERVLVTTLTKRMAEDLTEYLEEHGERVRYLHSDIDTVERMEIIRDLRLGEF
DVLVGINLLREGLDMPEVSLVAILDADKEGFLRSERSLIQTIGRAARNVNGKAILYGDKITPSMAKAIGET
ERRREKQQKYNEEHGITPQGLNKKVVDILALGQNIAKTKAKGRGKSRPIVEPDNVPMDMSPKALQQKIHEL
EGLMMQHAQNLEFEEAAQIRDQLHQLRELFIAAS
```

DBP5 in yeast is a cytoplasmic ATP-dependent RNA helicase of the DEAD-box family, e.g. involved in mRNA export from the nucleus or translation termination. The E. coli protein UvrB is a subunit of the nucleotide excision repair (NER) complex, which is responsible for repair of an assortment of DNA lesions (http://biocyc.org/ECOLI/NEW-IMAGE?type=ENZYME&object=EG11062-MONOMER).

673 aa (bacteria) vs. 482 aa (yeast)

DBP8

```
>DBP8|Escherichia_coli_str._K-12_substr._MG1655|AAC75803.1
MEPFKYICHYWGKSSKSLTKGNDIHLLIYHCLDVAAVADCWWDQSVVLQNTFCRNEMLSKQRVKAWLLFFI
ALHDIGKFDIRFQYKSAESWLKLNPATPSLNGPSTQMCRKFNHGAAGLYWFNQDSLSEQSLGDFFSFFDAA
PHPYESWFPWVEAVTGHHGFILHSQDQDKSRWEMPASLASYAAQDKQAREEWISVLEALFLTPAGLSINDI
PPDCSSLLAGFCSLADWLGSWTTTNTFLFNEDAPSDINALRTYFQDRQQDASRVLELSGLVSNKRCYEGVH
ALLDNGYQPRQLQVLVDALPVAPGLTVIEAPTGSGKTETALAYAWKLIDQQIADSVIFALPTQATANAMLT
RMEASASHLFSSPNLILAHGNSRFNHLFQSIKSRAITEQGQEEAWVQCCQWLSQSNKKVFLGQIGVCTIDQ
VLISVLPVKHRFIRGLGIGRSVLIVDEVHAYDTYMNGLLEAVLKAQADVGGSVILLSATLPMKQKQKLLDT
YGLHTDPVENNSAYPLINWRGVNGAQRFDLLAHPEQLPPRFSIQPEPICLADMLPDLTMLERMIAAANAGA
QVCLICNLVDVAQVCYQRLKELNNTQVDIDLFHARFTLNDRREKENRVISNFGKNGKRNVGRILVATQVVE
QSLDVDFDWLITQHCPADLLFQRLGRLHRHHRKYRPAGFEIPVATILLPDGEGYGRHEHIYSNVRVMWRTQ
QHIEELNGASLFFPDAYRQWLDSIYDDAEMDEPEWVGNGMDKFESAECEKRFKARKVLQWAEEYSLQDNDE
TILAVTRDGEMSLPLLPYVQTSSGKQLLDGQVYEDLSHEQQYEALALNRVNVPFTWKRSFSEVVDEDGLLW
LEGKQNLDGWVWQGNSIVITYTGDEGMTRVIPANPK
```

The yeast DBP8 is an ATPase, a RNA helicase of the DEAD-box family and a component of 90S preribosome complex involved in production of 18S rRNA and assembly of 40S small ribosomal subunit. In E.coli Cas3/YgcB is a protein involved in CRISPR R-loop formation and dissociation and is thus a Helicase as well. Cas3 catalyses <u>ATP-independent</u> annealing of RNA with DNA to form hybrid molecules of RNA base-paired into duplex DNA, also known as R-loops (Howard11: Howard JA, Delmas S, Ivancic-Bace I, Bolt EL (2011). "Helicase dissociation and annealing of RNA-DNA hybrids by Escherichia coli Cas3 protein." Biochem J 439(1);85-95. PMID: 21699496).

888 aa (bacteria) vs. 431 aa (yeast)

DED1

```
>DED1|Escherichia_coli_str._K-12_substr._MG1655|AAC76676.1
MKGRLLDAVPLSSLTGVGAALSNKLAKINLHTVQDLLLHLPLRYEDRTHLYPIGELLPGVYATVEGEVLNC
NISFGGRRMMTCQISDGSGILTMRFFNFSAAMKNSLAAGRRVLAYGEAKRGKYGAEMIHPEYRVQGDLSTP
ELQETLTPVYPTTEGVKQATLRKLTDQALDLLDTCAIEELLPPELSQGMMTLPEALRTLHRPPPTLQLSDL
ETGQHPAQRRLILEELLAHNLSMLALRAGAQRFHAQPLSANDTLKNKLLAALPFKPTGAQARVVAEIERDM
ALDVPMMRLVQGDVGSGKTLVAALAALRAIAHGKQVALMAPTELLAEQHANNFRNWFAPLGIEVGWLAGKQ
KGKARLAQQEAIASGQVQMIVGTHAIFQEQVQFNGLALVIIDEQHRFGVHQRLALWEKGQQQGFHPHQLIM
TATPIPRTLAMTAYADLDTSVIDELPPGRTPVTTVAIPDTRRTDIIDRVHHACITEGRQAYWVCTLIEESE
LLEAQAAEATWEELKLALPELNVGLVHGRMKPAEKQAVMASFKQGELHLLVATTVIEVGVDVPNASLMIIE
NPERLGLAQLHQLRGRVGRGAVASHCVLLYKTPLSKTAQIRLQVLRDSNDGFVIAQKDLEIRGPGELLGTR
QTGNAEFKVADLLRDQAMIPEVQRLARHIHERYPQQAKALIERWMPETERYSNA
```

DED1 is a ATP-dependent DEAD-box RNA helicase in yeast, while RecG is a DNA helicase involved in double-strand break repair and protecting against aberrant DNA replication following DNA damage (e.g. Rudolph CJ, Mahdi AA, Upton AL, Lloyd RG. RecG protein and single-strand DNA exonucleases avoid cell lethality associated with PriA helicase activity in Escherichia coli. Genetics. 2010 Oct;186(2):473-92.).

693 aa (bacteria) vs. 604 aa (yeast)

DIM1

```
>DIM1|Escherichia_coli_str._K-12_substr._MG1655|AAC73162.1
MNNRVHQGHLARKRFGQNFLNDQFVIDSIVSAINPQKGQAMVEIGPGLAALTEPVGERLDQLTVIELDRDL
AARLQTHPFLGPKLTIYQQDAMTFNFGELAEKMGQPLRVFGNLPYNISTPLMFHLFSYTDAIADMHFMLQK
EVVNRLVAGPNSKAYGRLSVMAQYYCNVIPVLEVPPSAFTPPPKVDSAVVRLVPHATMPHPVKDVRVLSRI
TTEAFNQRRKTIRNSLGNLFSVEVLTGMGIDPAMRAENISVAQYCQMANYLAENAPLQES
```

DIM1 is an essential 18S rRNA dimethylase (dimethyladenosine transferase), responsible for conserved m6(2)Am6(2)A dimethylation in the terminal loop in helix 45 of 18S rRNA, part of 90S and 40S pre-particles in nucleolus, involved in pre-ribosomal RNA processing. In E.coli RsmA/KsgA is the methyltransferase responsible for dimethylation of 16S rRNA at the two adjacent adenosine bases A1518 and A1519 (http://biocyc.org/ECOLI/NEW-IMAGE?type=ENZYME&object=EG10523-MONOMER).

273 aa (bacteria) vs. 318 aa (yeast)

DIS3

```
>DIS3|Escherichia_coli_str._K-12_substr._MG1655|AAC74368.1
MFQDNPLLAQLKQQLHSQTPRAEGVVKATEKGFGFLEVDAQKSYFIPPPQMKKVMHGDRIIAVIHSEKERE
SAEPEELVEPFLTRFVGKVQGKNDRLAIVPDHPLLKDAIPCRAARGLNHEFKEGDWAVAEMRRHPLKGDRS
FYAELTQYITFGDDHFVPWWVTLARHNLEKEAPDGVATEMLDEGLVREDLTALDFVTIDSASTEDMDDALF
AKALPDDKLQLIVAIADPTAWIAEGSKLDKAAKIRAFTNYLPGFNIPMLPRELSDDLCSLRANEVRPVLAC
RMTLSADGTIEDNIEFFAATIESKAKLVYDQVSDWLENTGDWQPESEAIAEQVRLLAQICQRRGEWRHNHA
LVFKDRPDYRFILGEKGEVLDIVAEPRRIANRIVEEAMIAANICAARVLRDKLGFGIYNVHMGFDPANADA
LAALLKTHGLHVDAEEVLTLDGFCKLRRELDAQPTGFLDSRIRRFQSFAEISTEPGPHFGLGLEAYATWTS
PIRKYGDMINHRLLKAVIKGETATRPQDEITVQMAERRRLNRMAERDVGDWLYARFLKDKAGTDTRFAAEI
VDISRGGMRVRLVDNGAIAFIPAPFLHAVRDELVCSQENGTVQIKGETVYKVTDVIDVTIAEVRMETRSII
ARPVA
```

The Dis3 is a exosome core complex catalytic subunit; possesses both endonuclease and 3'-5' exonuclease activity; involved in 3'-5' RNA processing and degradation in both the nucleus and the cytoplasm; has similarity to E. coli RNase R (rnr) and to human DIS3. E.coli RNB is RNAse II, which is closely related to RNAse R, Ribonuclease II (RNase II) is an exonuclease that cleaves RNA from the 3' end to produce ribonucleoside 5'-monophosphates.

644 aa (bacteria) vs. 1001 aa (yeast)

DRS1

```
>DRS1|Escherichia_coli_str._K-12_substr._MG1655|AAC74425.1
MTAFSTLNVLPPAQLTNLNELGYLTMTPVQAAALPAILAGKDVRVQAKTGSGKTAAFGLGLLQQIDASLFQ
TQALVLCPTRELADQVAGELRRLARFLPNTKILTLCGGQPFGMQRDSLQHAPHIIVATPGRLLDHLQKGTV
SLDALNTLVMDEADRMLDMGFSDAIDDVIRFAPASRQTLLFSATWPEAIAAISGRVQRDPLAIEIDSTDAL
PPIEQQFYETSSKGKIPLLQRLLSLHQPSSCVVFCNTKKDCQAVCDALNEVGQSALSLHGDLEQRDRDQTL
VRFANGSARVLVATDVAARGLDIKSLELVVNFELAWDPEVHVHRIGRTARAGNSGLAISFCAPEEAQRANI
ISDMLQIKLNWQTPPANSSIATLEAEMATLCIDGGKKAKMRPGDVLGALTGDIGLDGADIGKIAVHPAHVY
VAVRQAVAHKAWKQLQGGKIKGKTCRVRLLK
```

The *E.coli* protein DbpA is a ATP-dependent RNA helicase, specific for 23S rRNA and thus acts in the same pathway as the nucleolar DEAD-box DRS1 required for ribosome assembly and function, including synthesis of 60S ribosomal subunits.

457 aa (bacteria) vs. 752 aa (yeast)

FPR3

```
>FPR3|Escherichia_coli_str._K-12_substr._MG1655|AAC76372.1
MKSLFKVTLLATTMAVALHAPITFAAEAAKPATAADSKAAFKNDDQKSAYALGASLGRYMENSLKEQEKLG
IKLDKDQLIAGVQDAFADKSKLSDQEIEQTLQAFEARVKSSAQAKMEKDAADNEAKGKEYREKFAKEKGVK
```

TSSTGLVYQVVEAGKGEAPKDSDTVVVNYKGTLIDGKEFDNSYTRGEPLSFRLDGVIPGWTEGLKNIKKGG
KIKLVIPPELAYGKAGVPGIPPNSTLVFDVELLDVKPAPKADAKPEADAKAADSAKK

FPR3 in yeast is a Nucleolar peptidyl-prolyl cis-trans isomerase (PPIase), which is comparable to E. coli FkpA which is a FKBP-type peptidyl-prolyl cis-trans isomerase (rotamase)

270 aa (bacteria) vs. 411 aa (yeast)

KRE33
>KRE33|Escherichia_coli_str._K-12_substr._MG1655|AAC75527.1
MAELTALHTLTAQMKREGIRRLLVLSGEEGWCFEHTLKLRDALPGDWLWISPRPDAENHCSPSALQTLLGR
EFRHAVFDARHGFDAAAFAALSGTLKAGSWLVLLLPVWEEWENQPDADSLRWSDCPDPIATPHFVQHLKRV
LTADNEAILWRQNQPFSLAHFTPRTDWYPATGAPQPEQQQLLKQLMTMPPGVAAVTAARGRGKSALAGQLI
SRIAGRAIVTAPAKASTDVLAQFAGEKFRFIAPDALLASDEQADWLVVDEAAAIPAPLLHQLVSRFPRTLL
TTTVQGYEGTGRGFLLKFCARFPHLHRFELQQPIRWAQGCPLEKMVSEALVFDDENFTHTPQGNIVISAFE
QTLWQSDPETPLKVYQLLSGAHYRTSPLDLRRMMDAPGQHFLQAAGENEIAGALWLVDEGGLSQQLSQAVW
AGFRRPRGNLVAQSLAAHGNNPLAATLRGRRVSRIAVHPARQREGTGRQLIAGALQYTQDLDYLSVSFGYT
GELWRFWQRCGFVLVRMGNHREASSGCYTAMALLPMSDAGKQLAEREHYRLRRDAQALAQWNGETLPVDPL
NDAVLSDDDWLELAGFAFAHRPLLTSLGCLLRLLQTSELALPALRGRLQKNASDAQLCTTLKLSGRKMLLV
RQREEAAQALFALNDVRTERLRDRITQWQLFH

Kre33 is an essential protein, required for biogenesis of the small ribosomal subunit, but at stage it is not clear what it does. TmcA in E. coli is a elongator methionine tRNA (ac4C34) acetyltransferase. A crystal structure of the enzyme complexed with acetyl-CoA and ADP has been solved at 2.35 Å resolution. A modified DEAD-box RNA helicase module is formed by the peripheral N-terminal and the DUF699 PFAM domains. The authors propose that the RNA helicase motor delivers the wobble base substrate of the tRNA to the enzyme active site (Chimnaronk09: Chimnaronk S, Manita T, Ikeuchi Y, Yao M, Suzuki T, Tanaka I (2009). "RNA helicase module in an acetyltransferase that modifies a specific tRNA anticodon." EMBO J 28(9);1362-73.).

671 aa (bacteria) vs. 1056 aa (yeast)

MDN1
>MDN1|Escherichia_coli_str._K-12_substr._MG1655|AAC75180.2
MSPQNNHLQRPPAAVLYADELAKLKQNDNAPCPPGWQLSLPAARAFILGDEAQNISRKVVISPSAVERMLV
TLATGRGLMLVGEPGTAKSLLSELLATAISGDAGLTIQGGASTTEDQIKYGWNYALLINHGPSTEALVPAP
LYQGMRDGKIVRFEEITRTPLEVQDCLLGMLSDRVMTGPELTGEASQLYAREGFNIIATANTRDRGVNEMS
AALKRRFDFETVFPIMDFAQELELVASASARLLAHSGIPHKVPDAVLELLVRTFRDLRANGEKKTSMDTLT
AIMSTAEAVNVAHAVGVRAWFLANRAGEPADLVECIAGTIVKDNEEDRARLRRYFEQRVATHKEAHWQAYY
QARHRLP

The yeast MDN1 is a huge dynein-related AAA-type ATPase (midasin), which forms extended pre-60S particle with the Rix1 complex (Rix1p-Ipi1p-Ipi3p) and acts in removal of ribosomal biogenesis factors at successive steps of pre-60S assembly and export from nucleus. YehL defines a subfamily of the MoxR AAA+ ("ATPases associated with various cellular activities") family, but nothing is known, however, considering that E.coli protein is 362 amino acids and the yeast protein 4.910 amino acids, thus, it is hard to believe that they are orthologs.

362 aa (bacteria) vs. 4910 aa (yeast)

MTR4

```
>MTR4|Escherichia_coli_str._K-12_substr._MG1655|AAC75675.1
MTEIYEQAKHSLQGEDFSSFNYLFAVNKLLSNPVSYDLGRDLIVRALDSRERFSEHTTILKNMVRKSGLFP
YLKKEFTSLTPDDLRVLELYRTPFSDGYVFHSMQFHIFDLLKSGQNVVLSAPTSMGKSAIVDSLLGMGTLK
RLVLVVPTVALADETRRRLQERFGDRYQIIHHSSQVCHSDQAVYVLTQERVNERDDIVDIDLFVIDEFYKL
AFRQLKSGDIDHQDERVIELNIALSKLLKVSRQFYLTGPFVNSIRGLEKLGYPHTFVSTDFNTVALDVKTF
GIKANDDKAKLKALGEIAHACVDATIIYCKSPTVAGLVARELIRLGHGTPTENPHVDWVSEEFDADWDYTV
ALRNGIGLHFGALPRALQQYTADQFNAGKLRFLLCTSTIIEGVNTIAKNVVIYDNRDGTRSIDKFTHGNIK
GRAGRMGVHFVGKIFCLEEIPEDNLNQEVDIPLGIQGIDTPINLLASVQPDHLSEFSQDRFDEVFINDRVS
IDLVKKHSYFRVEQFEMLQSMFEMMDDNEFSSLVFHWTPATNFLKTFAKIIARLVPHTFSRNGVPVKPTDV
MIAKLAGYLSAESYSEYLKNQIDYARQWISEGEKRTLSIALNNDLKLITNTFGYTLPKVLSLMEDVVKHHA
VKRGIRSKVDYTHVKLAFESFHLPPGVNALEEIGIPIQTLHRLVDLLEFSDEADVDELSQYLRDTQDIWSR
SIGYVDQMFIRRALGIRRH
```

MTR4 is a ATP-dependent 3'-5' RNA helicase of the DExD/H family and involved in nuclear RNA processing and degradation. The E. coli protein yfjK is not described but shows a helicase signature.

726 aa (bacteria) vs. 1073 aa (yeast)

NOP2

```
>NOP2|Escherichia_coli_str._K-12_substr._MG1655|AAC74905.2
MAQHTVYFPDAFLTQMREAMPSTLSFDDFLAACQRPLRRSIRVNTLKISVADFLQLTAPYGWTLTPIPWCE
EGFWIERDNEDALPLGSTAEHLSGLFYIQEASSMLPVAALFADGNAPQRVMDVAAAPGSKTTQISARMNNE
GAILANEFSASRVKVLHANISRCGISNVALTHFDGRVFGAAVPEMFDAILLDAPCSGEGVVRKDPDALKNW
SPESNQEIAATQRELIDSAFHALRPGGTLVYSTCTLNQEENEAVCLWLKETYPDAVEFLPLGDLFPGANKA
LTEEGFLHVFPQIYDCEGFFVARLRKTQAIPALPAPKYKVGNFPFSPVKDREAGQIRQAATGVGLNWDENL
RLWQRDKELWLFPVGIEALIGKVRFSRLGIKLAETHNKGYRWQHEAVIALASPDNMNAFELTPQEAEEWYR
GRDVYPQAAPVADDVLVTFQHQPIGLAKRIGSRLKNSYPRELVRDGKLFTGNA
```

NOP2 is a RNA m(5)C methyltransferase, which is essential for processing and maturation of 27S pre-rRNA and large ribosomal subunit biogenesis and which is localized to the nucleolus. The E. coli protein RsmF is a SAM-dependent 16S rRNA m(5)C1407 methyltransferase.

476 aa (bacteria) vs. 618 aa (yeast)

RCL1

```
>RCL1|Escherichia_coli_str._K-12_substr._MG1655|AAT48181.1
MKRMIALDGAQGEGGGQILRSALSLSMITGQPFTITSIRAGRAKPGLLRQHLTAVKAATEICGATVEGAEL
GSQRLLFRPGTVRGGDYRFAIGSAGSCTLVLQTVLPALWFADGPSRVEVSGGTDNPSAPPADFIRRVLEPL
LAKIGIHQQTTLLRHGFYPAGGGVVATEVSPVASFNTLQLGERGNIVQMRGEVLLAGVPRHVAEREIATLA
GSFSLHEQNIHNLPRDQGPGNTVSLEVESENITERFFVVGEKRVSAEVVAAQLVKEVKRYLASTAAVGEYL
ADQLVLPMALAGAGEFTVAHPSCHLLTNIAVVERFLPVRFSLIETDGVTRVSIE
```

RCL1 is a endonuclease suggested to cleave pre-rRNA at site A2 for 18S rRNA biogenesis, it is a subunit of U3-containing 90S preribosome processome complex, the E.coli RtcA is a RNA 3'-terminal phosphate cyclase of unknown physiological function

338 aa (bacteria) vs. 367 aa (yeast)

REX2

>REX2|Escherichia_coli_str._K-12_substr._MG1655|AAC77122.2
MSANENNLIWIDLEMTGLDPERDRIIEIATLVTDANLNILAEGPTIAVHQSDEQLALMDDWNVRTHTASGL
VERVKASTMGDREAELATLEFLKQWVPAGKSPICGNSIGQDRRFLFKYMPELEAYFHYRYLDVSTLKELAR
RWKPEILDGFTKQGTHQAMDDIRESVAELAYYREHFIKL

Rex2 is a 3'-5' RNA exonuclease which is involved e.g. in 3'-end processing of U4 and U5 snRNAs. The oligoribonuclease YjeR (Orn) is a processive 3'-to-5' exoribonuclease specific for short oligoribonucleotides.

181 aa (bacteria) vs. 269 aa (yeast)

RIX7
>RIX7|Escherichia_coli_str._K-12_substr._MG1655|AAC74930.1
MIEADRLISAGTTLPEDVADRAIRPKLLEEYVGQPQVRSQMEIFIKAAKLRGDALDHLLIFGPPGLGKTTL
ANIVANEMGVNLRTTSGPVLEKAGDLAAMLTNLEPHDVLFIDEIHRLSPVVEEVLYPAMEDYQLDIMIGEG
PAARSIKIDLPPFTLIGATTRAGSLTSPLRDRFGIVQRLEFYQVPDLQYIVSRSARFMGLEMSDDGALEVA
RRARGTPRIANRLLRRVRDFAEVKHDGTISADIAAQALDMLNVDAEGFDYMDRKLLLAVIDKFFGGPVGLD
NLAAAIGEERETIEDVLEPYLIQQGFLQRTPRGRMATTRAWNHFGITPPEMP

Rix7 is a putative ATPase of the AAA family, required for export of pre-ribosomal large subunits from the nucleus, while RuvB is ATP-dependent DNA helicase, component of RuvABC resolvasome

336 aa (bacteria) vs. 837 aa (yeast)

RLI1
>RLI1|Escherichia_coli_str._K-12_substr._MG1655|AAC74700.1
MNAIWIAVAAVSLLGLAFGAILGYASRRFAVEDDPVVEKIDEILPQSQCGQCGYPGCRPYAEAISCNGEKI
NRCAPGGEAVMLKIAELLNVEPQPLDGEAQEITPARMVAVIDENNCIGCTKCIQACPVDAIVGATRAMHTV
MSDLCTGCNLCVDPCPTHCISLQPVAETPDSWKWDLNTIPVRIIPVEHHA

RLI1 in yeast is an essential iron-sulfur protein required for ribosome biogenesis and translation initiation and termination, the E coli rsxB is an iron-sulfur cluster containing electron transport complex protein required for the reduction of SoxR (Koo MS, Lee JH, Rah SY, Yeo WS, Lee JW, Lee KL, Koh YS, Kang SO, Roe JH (2003). "A reducing system of the superoxide sensor SoxR in Escherichia coli." EMBO J 22;2614-22.)

192 aa (bacteria) vs. 608 aa (yeast)

RNT1
>RNT1|Escherichia_coli_str._K-12_substr._MG1655|AAC75620.1
MNPIVINRLQRKLGYTFNHQELLQQALTHRSASSKHNERLEFLGDSILSYVIANALYHRFPRVDEGDMSRM
RATLVRGNTLAELAREFELGECLRLGPGELKSGGFRRESILADTVEALIGGVFLDSDIQTVEKLILNWYQT
RLDEISPGDKQKDPKTRLQEYLQGRHLPLPTYLVVQVRGEAHDQEFTIHCQVSGLSEPVVGTGSSRRKAEQ
AAAEQALKKLELE

RNT1 is RNAase III, which is involved in rDNA transcription termination and rRNA processing, while RNC is the RNase IIIof E coli and required for processing of ribosomal RNA (rRNA).

226 aa (bacteria) vs. 471 aa (yeast)

ROX1

>ROK1|Escherichia_coli_str._K-12_substr._MG1655|AAC74198.1
MPEQYRYTLPVKAGEQRLLGELTGAACATLVAEIAERHAGPVVLIAPDMQNALRLHDEISQFTDQMVMNLA
DWETLPYDSFSPHQDIISSRLSTLYQLPTMQRGVLIVPVNTLMQRVCPHSFLHGHALVMKKGQRLSRDALR
TQLDSAGYRHVDQVMEHGEYATRGALLDLFPMGSELPYRLDFFDDEIDSLRVFDVDSQRTLEEVEAINLLP
AHEFPTDKAAIELFRSQWRDTFEVKRDPEHIYQQVSKGTLPAGIEYWQPLFFSEPLPPLFSYFPANTLLVN
TGDLETSAERFQADTLARFENRGVDPMRPLLPPQSLWLRVDELFSELKNWPRVQLKTEHLPTKAANANLGF
QKLPDLAVQAQQKAPLDALRKFLETFDGPVVFSVESEGRREALGELLARIKIAPQRIMRLDEASDRGRYLM
IGAAEHGFVDTVRNLALICESDLLGERVARRRQDSRRTINPDTLIRNLAELHIGQPVVHLEHGVGRYAGMT
TLEAGGITGEYLMLTYANDAKLYVPVSSLHLISRYAGGAEENAPLHKLGGDAWSRARQKAAEKVRDVAAEL
LDIYAQRAAKEGFAFKHDREQYQLFCDSFPFETTPDQAQAINAVLSDMCQPLAMDRLVCGDVGFGKTEVAM
RAAFLAVDNHKQVAVLVPTTLLAQQHYDNFRDRFANWPVRIEMISRFRSAKEQTQILAEVAEGKIDILIGT
HKLLQSDVKFKDLGLLIVDEEHRFGVRHKERIKAMRANVDILTLTATPIPRTLNMAMSGMRDLSIIATPPA
RRLAVKTFVREYDSMVVREAILREILRGGQVYYLYNDVENIQKAAERLAELVPEARIAIGHGQMRERELER
VMNDFHHQRFNVLVCTTIIETGIDIPTANTIIIERADHFGLAQLHQLRGRVGRSHHQAYAWLLTPHPKAMT
TDAQKRLEAIASLEDLGAGFALATHDLEIRGAGELLGEEQSGSMETIGFSLYMELLENAVDALKAGREPSL
EDLTSQQTEVELRMPSLLPDDFIPDVNTRLSFYKRIASAKTENELEEIKVELIDRFGLLPDPARTLLDIAR
LRQQAQKLGIRKLEGNEKGGVIEFAEKNHVNPAWLIGLLQKQPQHYRLDGPTRLKFIQDLSERKTRIEWVR
QFMRELEENAIA

Rok1 is a RNA-dependent ATPase, which is required for pre-rRNA processing at sites A0, A1, and A2, and in control of cell cycle progression. MFD in E coli is responsible for ATP-dependent removal of stalled RNA polymerase from DNA lesions.

1148 aa (bacteria) vs.564 aa (yeast)

RRP5
>RRP5|Escherichia_coli_str._K-12_substr._MG1655|AAC73997.1
MTESFAQLFEESLKEIETRPGSIVRGVVVAIDKDVVLVDAGLKSESAIPAEQFKNAQGELEIQVGDEVDVA
LDAVEDGFGETLLSREKAKRHEAWITLEKAYEDAETVTGVINGKVKGGFTVELNGIRAFLPGSLVDVRPVR
DTLHLEGKELEFKVIKLDQKRNNVVVSRRAVIESENSAERDQLLENLQEGMEVKGIVKNLTDYGAFVDLGG
VDGLLHITDMAWKRVKHPSEIVNVGDEITVKVLKFDRERTRVSLGLKQLGEDPWVAIAKRYPEGTKLTGRV
TNLTDYGCFVEIEEGVEGLVHVSEMDWTNKNIHPSKVVNVGDVVEVMVLDIDEERRRISLGLKQCKANPWQ
QFAETHNKGDRVEGKIKSITDFGIFIGLDGGIDGLVHLSDISWNVAGEEAVREYKKGDEIAAVVLQVDAER
ERISLGVKQLAEDPFNNWVALNKKGAIVTGKVTAVDAKGATVELADGVEGYLRASEASRDRVEDATLVLSV
GDEVEAKFTGVDRKNRAISLSVRAKDEADEKDAIATVNKQEDANFSNNAMAEAFKAAKGE

RRP5 is a RNA binding protein with preference for single stranded tracts of U's involved in synthesis of both 18S and 5.8S rRNAs and is a component of both the ribosomal small subunit (SSU) processosome and the 90S preribosome. RPSA is also termed 30S ribosomal subunit protein S1 and thus a component of the ribosome. As RPSA is significantly smaller than RRP5, it might only represent an equivalent domain only

557 aa (bacteria) vs. 1729 aa (yeast)

RRP6
>RRP6|Escherichia_coli_str._K-12_substr._MG1655|AAC74874.1
MNYQMITTDDALASLCEAVRAFPAIALDTEFVRTRTYYPQLGLIQLFDGEHLALIDPLGITDWSPLKAILR
DPSITKFLHAGSEDLEVFLNVFGELPQPLIDTQILAAFCGRPMSWGFASMVEEYSGVTLDKSESRTDWLAR
PLTERQCEYAAADVWYLLPITAKLMVETEASGWLPAALDECRLMQMRRQEVVAPEDAWRDITNAWQLRTRQ
LACLQLLADWRLRKARERDLAVNFVVREEHLWSVARYMPGSLGELDSLGLSGSEIRFHGKTLLALVEKAQT
LPEDALPQPMLNLMDMPGYRKAFKAIKSLITDVSETHKISAELLASRRQINQLLNWHWKLKPQNNLPELIS
GWRGELMAEALHNLLQEYPQ

The Ecoli protein RND is the ribonuclease D, an exonuclease involved in the 3' ribonucleolytic processing of precursor tRNA. RRP6 is a nuclear exosome exonuclease component which has 3'-5' exonuclease activity and is involved in RNA processing, maturation, surveillance, degradation, tethering, and export. It was described to have similarity to E. coli RNase D (Midtgaard SF, Assenholt J, Jonstrup AT, Van LB, Jensen TH, Brodersen DE. Structure of the nuclear exosome component Rrp6p reveals an interplay between the active site and the HRDC domain. Proc Natl Acad Sci U S A. 2006;103(32):11898-903; Zuo Y, Wang Y, Malhotra A. Crystal structure of Escherichia coli RNase D, an exoribonuclease involved in structured RNA processing. Structure. 2005 Jul;13(7):973-84.);

375 aa (bacteria) vs. 733 aa (yeast)

SKI6

```
>SKI6|Escherichia_coli_str._K-12_substr._MG1655|ABV59576.1
MRPAGRSNNQVRPVTLTRNYTKHAEGSVLVEFGDTKVLCTASIEEGVPRFLKGQGQGWITAEYGMLPRSTH
TRNAREAAKGKQGGRTMEIQRLIARALRAAVDLKALGEFTITLDCDVLQADGGTRTASITGACVALVDALQ
KLVENGKLKTNPMKGMVAAVSVGIVNGEAVCDLEYVEDSAAETDMNVVMTEDGRIIEVQGTAEGEPFTHEE
LLILLALARGESNPL
```

Ski6 is a non-catalytic core component of the exosome,is involved in 3'-5' RNA processing and degradation in both the nucleus and the cytoplasm, which has similarity to E. coli RNase PH (Ski6p is a homolog of RNA-processing enzymes that affects translation of non-poly(A) mRNAs and 60S ribosomal subunit biogenesis. Benard, L., Carroll, K., Valle, R.C., Wickner, R.B. Mol. Cell. Biol. (1998)).The E coli protein RPH is the RNase PH.

228 aa (bacteria) vs. 246 aa (yeast)

SUMMARY

	Yeast		E.coli	
	Name	aa	Name	aa
**1**	**ARB1**	**610**	**YbhF**	**578**
2	BUD23	275	BioC	251
3	CDC14	551	*YnbD*	430
4	CFD1	293	*ApbC*	369
5	DBP5	482	*UvrB*	673
6	DBP8	431	*Cas3*	888
**7**	**DED1**	**604**	**RecG**	**693**
**8**	**DIM1**	**318**	**RsmA**	**273**
**9**	**DIS3**	**1001**	**RNB**	**644**
**10**	**DRS1**	**752**	**DbpA**	**457**
**11**	**FPR3**	**411**	**FkpA**	**270**
12	Kre33	1056	TmcA	671
13	MDN1	4910	YehL	362
**14**	**MTR4**	**1073**	**YfjK**	**726**
**15**	**NOP2**	**618**	**RsmF**	**476**
**16**	**RCL1**	**367**	**RtcA**	**338**
**17**	**REX2**	**269**	**YjeR**	**181**
18	RIX7	837	RuvB	336
19	RLI1	608	RsxB	192
**20**	**RNT1**	**471**	**RNC**	**226**
21	ROK1	564	MFD	1148
**22**	**RRP5**	**1729**	**RPSA**	**557**
**23**	**RRP6**	**733**	**RND**	**375**
**24**	**SKI6**	**246**	**RPH**	**228**

Factors in bold face are likely to possess a comparable activity in yeast and Bacteria.
